# Coupling internal cerebellar models enhances online adaptation and supports offline consolidation in sensorimotor tasks

**DOI:** 10.3389/fncom.2013.00095

**Published:** 2013-07-15

**Authors:** Jean-Baptiste Passot, Niceto R. Luque, Angelo Arleo

**Affiliations:** ^1^Unit of Neurobiology of Adaptive Processes, Centre National de la Recherche Scientifique, UPMC University Paris 6, UMR 7102Paris, France; ^2^UGR – Computer Architecture and Technology Department, University of GranadaGranada, Spain

**Keywords:** cerebellar microcomplex, sensorimotor adaptation, inverse and forward internal models, procedural adaptation task, motor control

## Abstract

The cerebellum is thought to mediate sensorimotor adaptation through the acquisition of internal models of the body-environment interaction. These representations can be of two types, identified as forward and inverse models. The first predicts the sensory consequences of actions, while the second provides the correct commands to achieve desired state transitions. In this paper, we propose a composite architecture consisting of multiple cerebellar internal models to account for the adaptation performance of humans during sensorimotor learning. The proposed model takes inspiration from the cerebellar microcomplex circuit, and employs spiking neurons to process information. We investigate the intrinsic properties of the cerebellar circuitry subserving efficient adaptation properties, and we assess the complementary contributions of internal representations by simulating our model in a procedural adaptation task. Our simulation results suggest that the coupling of internal models enhances learning performance significantly (compared with independent forward and inverse models), and it allows for the reproduction of human adaptation capabilities. Furthermore, we provide a computational explanation for the performance improvement observed after one night of sleep in a wide range of sensorimotor tasks. We predict that internal model coupling is a necessary condition for the offline consolidation of procedural memories.

## 1. Introduction

The cerebellum plays a prominent role in motor control, movement coordination, and context-dependent sensorimotor adaptation (Ito, 2002; Fine et al., 2002; Ito, 2006). Fast and coordinated movements cannot be executed by relying on feedback information alone (Wolpert and Ghahramani, 2000; Shadmehr et al., 2010). Indeed, (1) neuronal nerves transmit information at relatively low speeds, which delays the sensory feedback necessary to adapt motor commands; (2) neural processing and integration of multimodal sensory feedback can require tens of milliseconds, thus preventing real-time sensorimotor adaptation; (3) sensory signals can be noisy, inaccurate, and often incomplete (e.g., the absence of visual feedback in darkness conditions). The internal model hypothesis postulates that the brain solves these limitations by acquiring internal representations of the body-world interaction (Ito, 1970, 1984; Kawato et al., 1987; McIntyre et al., 2001). Forward internal models predict the sensory outcomes of actions by estimating the causal relationship between sensory inputs and motor outputs (Goodwin, 1984; Ito, 1984; Miall et al., 1993; Wolpert and Miall, 1996). Inverse internal models work in the opposite direction, by estimating the motor commands that lead to desired state updates (Contreras-Vidal et al., 1997; Schweighofer et al., 1998; Kawato, 1999; Sethu Vijayakumar and Schaal, 2005). Both forward and inverse models depend on the dynamics of the motor system and must adapt to contextual changes as well as motor apparatus modifications (Lalazar and Vaadia, 2008). Experimental evidence from behavioural, functional imaging, and neurophysiological studies suggests that the cerebellum can acquire and store internal models mediating procedural learning (Bell et al., 1997; Miall, 1998; Wolpert et al., 1998; Eskandar and Assad, 1999; Kawato et al., 2003; Ito, 2005; Pasalar et al., 2006; Mulliken et al., 2008).

Several works considered what type of internal model can be plausibly implementable by the cerebellar anatomofunctional architecture (e.g., Pasalar et al., 2006). However, very few studies have evaluated the advantages of coupling internal models for online sensorimotor learning (Jordan and Rumelhart, 1992; Wolpert and Kawato, 1998). Furthermore, to the best of our knowledge, no study thus far has addressed the possible role of coupling internal cerebellar models for offline sensorimotor consolidation. Sleep is known to contribute to the enhancement of motor adaptation capabilities in humans (Walker et al., 2002; Huber et al., 2004; Stickgold, 2005). For instance, sleeping periods can lead to significant performance improvements not only in speed but also in accuracy on a sequential finger-tapping task, whereas equivalent awake periods do not provide significant benefits (Walker et al., 2002). This sleep-dependent motor adaptation enhancement and the well-known cerebellar implication in procedural learning (Thach et al., 1992; Martin et al., 1996; Imamizu et al., 2000; Kahlon and Lisberger, 2000; Kawato et al., 2003; Miall et al., 2007) raise the question of whether the functional coupling of forward and inverse cerebellar models may be relevant to the offline consolidation of sensorimotor memories following the execution of procedural tasks. Here, we address this question by validating a cerebellar coupling model for coordinated arm movement adaptation. In contrast to earlier coupling schemes (Jordan and Rumelhart, 1992; Wolpert and Kawato, 1998), we implement a spiking cerebellar neural network endowed with long-term synaptic plasticity. We validate the online and offline learning properties of the coupling model against human experimental data in a rotation adaptation task (Huber et al., 2004). Our simulations suggest that (and show how) the interplay between forward and inverse cerebellar models can enhance online sensorimotor adaptation to a level which is consistent with human learning performance. Furthermore, we show that the internal model coupling hypothesis can account for the significant performance increase exhibited by humans after one night of sleep (Huber et al., 2004).

## 2. Materials and methods

Here, we first describe the integrated control architecture for adaptive voluntary movements. Then, we focus on the connectivity layout and input–output function of the cerebellar microcomplex model used to implement both *forward predictors* and *inverse correctors* (more comprehensive accounts on neuronal model equations and parameter settings can be found in Supplementary Methods). Finally, we describe the simulated setup and protocol that reproduce the experiments by Huber et al. (2004), and we define the measures to assess sensorimotor adaptation.

### 2.1. Sensorimotor control architecture

#### 2.1.1. Integrated model for adaptive voluntary movements

**Figure 1:**
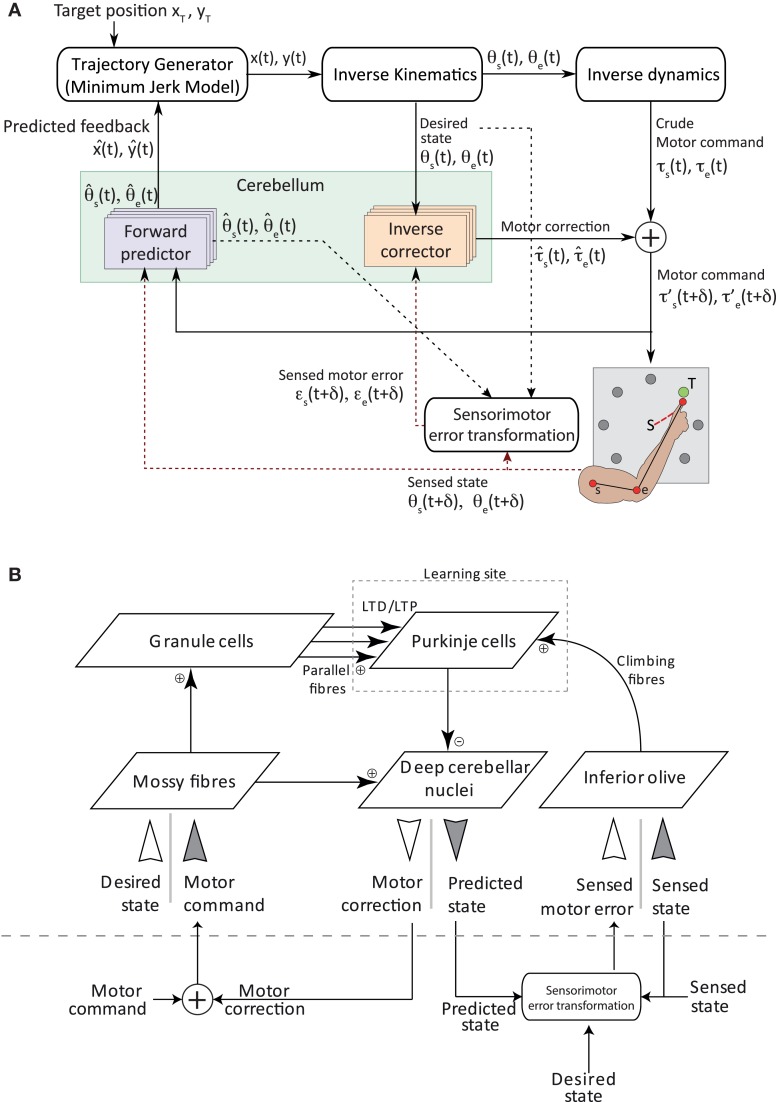
**Sensorimotor control architecture. (A)** Overview of the integrated model for adaptive voluntary movements. An optimal controller computes a set of torque commands to generate smooth trajectories of the arm end-point (i.e., the “finger”) toward target positions. Desired states of both joints reach a set of four inverse cerebellar models, which estimate the corrective torques used to produce the final motor commands. The cerebellar forward model uses the motor command efference copy to predict the future state (position and speed) of the arm end-point and sends it to the high-level controller. Feedback signals include state information (i.e., the angular position and speed of each joint) as well as motor error information (i.e., the difference between desired and actual state of each joint). These signals (drawn in red dash) provide the basis to compute the teaching signals for both cerebellar internal models. The sensorimotor error module is responsible for transforming a sensory error—using desired, predicted and sensed state, represented in black and red dash line—into a motor error. Motor commands and feedback signals are algorithmically delayed (both by δ = 50 ms in the simulations presented here) to account for central↔peripheral transmission delays in biological systems. The execution of each trajectory takes 0.7 s followed by a 0.3 s refractory period, during which joint positions are reset and cerebellar neuronal activities return to baseline level. We also assume that high-level recalculations of the entire arm trajectories cannot be performed in the absence of forward model predictions. **(B)** Model cerebellar microcomplex circuit. Each box indicates a population of spiking neurons. The same cerebellar circuit implements both forward (dark gray inputs) and inverse (white inputs) internal models.

Figure 1A shows the model architecture used to control a simulated 2 degree of freedom (2-DOF) arm in real time. The arm has two joints—one at the shoulder level (s) and one at the elbow level (e). A high-level (cortical-like) module generates the goal-oriented trajectories of the arm as well as the corresponding step-by-step motor commands (taken from Carrillo et al., 2008). This module is purely algorithmic [see Shadmehr et al. (2010) for a review of recent studies modeling motor cortex and basal ganglia functions].

At each time step Δ*t* (1 ms), a controller computes the torque commands τ_*s*_,τ_*e*_ for the shoulder and the elbow, respectively, in order to generate a smooth movement of the arm end-point (i.e., the finger) toward a target position (*x_T_, y_T_*). In addition, it provides the desired angular positions θ_*s*_,θ_*e*_. The high-level controller is composed of three modules serially connected: a trajectory generator, an inverse kinematics model and an inverse dynamics model. The trajectory generator uses a minimum jerk model (Flash and Hogan, 1985) to compute the desired smooth movement of the arm end-point toward the target position (*x_T_, y_T_*). The trajectory is expressed in Cartesian coordinates for each step of the movement. The desired movement is then transformed into arm-related coordinates, i.e., the desired angular position θ(*t*) = (θ_*s*_,θ_*e*_) for the shoulder and the elbow. These coordinates form the input to a crude inverse dynamics controller, which generates a set of torque commands τ(*t*) = (τ_*s*_,τ_*e*_). All mathematical solutions of minimum jerk, inverse kinematics and inverse dynamics models were taken from Carrillo et al. (2008) and are detailed in Supplementary Methods. This architecture was developed to study how internal models can improve the accuracy of fast reaching movements for which the delayed sensory feedback is unlikely to play a role in changing the dynamics of the movements. Hence, in our simulation, we make the assumption that the sensory feedback cannot be used to adapt the descending motor commands.

***Cerebellar-dependent inverse dynamics correction.*** The high-level controller sends the desired angular positions, θ_*s*_,θ_*e*_, to a set of cerebellar inverse corrector models that provide compensatory torque signals ${\hat{\tau }}_{s}$,${\hat{\tau }}_{e}$ to counter dynamical perturbations (e.g., shifts) occurring during movement execution. Four inverse correctors learn context-dependent motor command adaptation. Two of them compensate for movement execution errors of the shoulder by generating corrective torque commands ${\hat{\tau }}_{s}$ (positive and negative ranges, respectively, emulating agonist and antagonist muscles; Kumamoto et al., 1994). The other two generate elbow torque corrections ${\hat{\tau }}_{e}$ (positive and negative ranges, respectively). The final motor commands eventually sent to each arm joint are then ${\tau^{\prime }}_{s}=\tau_{s}+{\hat{\tau }}_{s}$, and ${\tau^{\prime }}_{e}=\tau_{e}+{\hat{\tau }}_{e}$ and are delayed by δ = 50 ms to account for central to peripheral transmission delays in biological systems (Figure 1A).

***Cerebellar-dependent forward state prediction.*** In parallel, four cerebellar forward predictors receive efference copies of the final torque commands τ′_*s*_, τ′_*e*_ and estimate the future angular position and velocity of each articulation, ${\hat{\theta }}_{s}(t)$, ${\hat{\dot{\theta }}}_{s}(t)$ and ${\hat{\theta }}_{e}(t)$, ${\hat{\dot{\theta }}}_{e}(t)$, respectively. In Figure 1A, ${\hat{\theta }}_{s/e}(t)$ represents the state of the joint at time *t* and comprises both the position and velocity of the joint. These forward state predictions are then algorithmically mapped onto arm end-point positions in Cartesian coordinates $\left(\hat{x},\hat{y}\right)$, and sent to the trajectory generator. The latter compares the desired and predicted position of the arm and consequently updates the trajectory (Figure 1A).

***Coupling cerebellar-dependent motor corrections and state predictions.*** Our model allows the relative importance of predictive and corrective sensorimotor signals to be evaluated. In a purely forward scheme, only the four cerebellar microcomplex models implementing internal forward predictors would be active and adapting over time. Conversely, in a purely inverse scheme, only the four cerebellar microcomplexes implementing inverse correctors would mediate sensorimotor adaptation. Under the coupling scheme, both inverse and forward models are active and learning. During online adaptation, the coupled system benefits at first from the fast learning dynamics of forward predictive models (see section 3). Based on the learnt forward predictions, the high-level controller can update the motor commands to account for predicted errors, which allows the resulting end-effector trajectory to be coarsely appropriate. In parallel, inverse corrector models learn to produce torque corrections to finely tune goal-oriented trajectories, which further improves the overall performance of the online adaptation process (see Supplementary Figure S1A for a functional view of the online coupling scheme). The teaching signal driving adaptation in forward predictor models corresponds to the actual state of each articulation, i.e., its angular position $\bar{\theta }=({\bar{\theta }}_{s},{\bar{\theta }}_{e})$ and velocity $\bar{\dot{\theta }}=({\bar{\dot{\theta }}}_{s},{\bar{\dot{\theta }}}_{e})$, post execution of each motor command. For inverse correctors, the teaching signal is derived from the difference between the desired and sensed angular positions and velocities, i.e., $\theta -\bar{\theta }$ and $\dot{\theta }-\bar{\dot{\theta }}$, for both joints (Figure 1A; Supplementary Methods S 1.6). The sensory feedback (the sensed position and velocity of each joint) are delayed by δ = 50 ms to account for peripheral to central transmission delays in biological systems (Figure 1A).

The coupling system also supports offline sensorimotor adaptation by driving the consolidation of procedural learning following the execution of a task. During processing in the absence of feedback signals, the state predictions learnt by the forward model during online adaptation can be used to train the inverse model offline. In this case, the teaching signal for the inverse correctors is a function of the difference between the desired and predicted angular state of each joint, (see Supplementary Figure S1B). This scheme assumes that entire action sequences, or at least desired states, can be replayed during offline processing. Experimental evidence suggesting that patterns of brain activity elicited during online training are subsequently replayed during sleep (Wilson and McNaughton, 1994; Kudrimoti et al., 1999; Maquet et al., 2000, 2003) supports this hypothesis (see section 4, for further details). During offline consolidation, the teaching signal driving adaptation in the forward predictors is absent, hence no modification is supposed to occur in forward predictors.

#### 2.1.2. The cerebellar microcomplex model

We model a simplified cerebellar microcomplex circuit (Figure 1B) capable of adapting its input–output dynamics through training. In agreement with Marr-Albus-Ito theory (Marr, 1969; Albus, 1971; Ito and Kano, 1982), we assume that the cerebellum can acquire internal models of complex sensorimotor interactions (Ito, 1970; Wolpert et al., 1998) and store them in multiple and coupled microcomplexes—the cerebellar computational units (Ito, 1984).

We model the basic elements of the cerebellar microcircuit by means of spiking neuronal network populations. Contextual input signals enter the network via the mossy fibres (MFs), which are connected by excitatory synapses to the granule cells (GCs) and to the deep cerebellar nuclei (DCN). Purkinje cells (PCs) receive excitatory inputs from both GCs (via the parallel fibres, PFs) and inferior olive (IO) neurons (via the climbing fibres, CFs). PCs inhibit the DCN cells, which are the output neurons of the microcircuit. A comprehensive account of the employed coding scheme is given in the Supplementary Methods and illustrated in Figures S2 and S3 for the inverse corrector and the forward predictor, respectively. Note that the encoding of the output is not the same for the forward and inverse corrective models in our model: a local code is employed to decode the output of the forward models whereas the mean firing rate of the population is used to decode the output of the inverse corrective models. Furthermore, the size of each module has been chosen so that an increase of the number of units does not lead to a significant an increase in performance of the simulated system (the size of each layer and connectivity are described in Supplementary Table A).

The basic learning principle in the neural network is the following: MFs excite DCN neurons via constant all-to-all connections. Hence, without inhibition from PCs, the output of such a defective microcircuit is constant and does not depend on the input. In order for the output to be meaningful, the strength of the inhibitory output of the PCs should depend on the input conveyed by MFs via GCs and PFs. The GC layer provides a sparse representation of the MF inputs—the number of GC neurons is 100 times larger than that of MFs and the MF–GC connection probability is only 0.04 (i.e., each MF innervates 400 GCs and each GC receives 4 MF afferents on average, in agreement with anatomical data (Eccles et al., 1967; Jakab and Hamori, 1988; Chadderton et al., 2004). A sparse representation serves to optimise the encoding capacity and information transmission from MFs to PCs (D'Angelo and De Zeeuw, 2009). The synapses between PFs (i.e., GCs' output fibres) and PCs are the only plastic synapses implemented in the microcircuit model, and they learn to translate the sparsely represented input into PC output that inhibits DCN. Bidirectional long-term plasticity (i.e., potentiation, LTP, and depression, LTD) modifies the efficacies of PF–PC synapses and shapes the input–output dynamics of the microcomplex. We implement LTP as a non-associative mechanism (Lev-Ram et al., 2002), such that every incoming PF spike triggers a synaptic weight increase. We model LTD as a supervised associative mechanism with the teaching signal conveyed by CFs (output fibres of the IO neurons). This is in accordance with experimental data showing that conjunctive inputs to a PC from PFs and CFs tend to depress PF–PC projections (Ito and Kano, 1982; Wang et al., 2000; Safo and Regehr, 2008).

### 2.2. Motor learning task and data analysis

**Figure 2:**
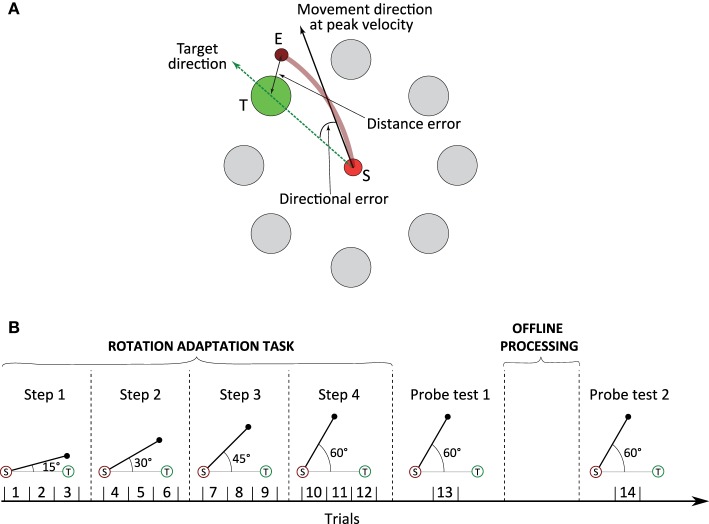
**Experimental setup and protocol (adapted from Huber et al., 2004). (A)** The rotation adaptation task includes eight targets (large circles) evenly distributed on a circle of centre *S*. Each hand movement initiates from the starting position *S* and must reach a target (green circle) randomly selected every second. Distance and directional errors are calculated when a subject stops its movement. The former error is the distance between the position of the target (T) and the final position of the hand when movement stops (E). The directional error is the angle between the line representing the target direction (dotted green line) and the line representing the movement at the peak outward velocity (solid black line). Both errors are normalised. For example, given an angular bias of 15°, a directional error equal to 1 in our simulation would correspond to an error of 15°, whereas a distance error equal to 1 would represent a distance of 36 mm between *E* and *T*. **(B)** Experimental protocol. Training involves 4 steps of 3 trials each (a trial lasts 90 s). At each step, we increase the angular bias by 15°, from 15° in step 1 to 60° in step 4. The extent of the rotation adaptation is tested after the 12 training trials by means of a first probe test involving an angular bias of 60° (trial 13). Then, a specific group of subjects (see main text) undergoes an offline consolidation process consisting of a series of 48 trials in offline mode. Finally, we evaluate the effect of the offline consolidation by means of a second probe test (trial 14) again with an angular bias of 60°.

We test the online and offline learning performance of the model in a simulated protocol that reproduces the *rotation adaptation task* carried out by Huber et al. (2004) on human subjects. It is defined by a central position *S* with eight targets evenly distributed on a circle centred on *S* (Figure 2A). Each arm end-point trajectory starts from *S* and aims at reaching one of the targets in a straight trajectory. In the simulated task, a new target is randomly chosen every 1 s. We emulate the rotational anticlockwise bias used by Huber et al. (2004) to force subjects to adapt their control policy (and thus compensate for the perceived trajectory errors) by adding a systematic angular deviation to the hand trajectory computed by the controller.

#### 2.2.1. Online sensorimotor adaptation

In the first phase of the simulated protocol, the angular bias evolves over time. There are four incremental steps, with the bias increasing by 15° at each step within the range [15°, 60°] (Figure 2B). Every step consists of three trials (one trial is the succession of 90 trajectories).

Three simulated groups of ten individuals each, undergo the rotation adaptation task. The “forward model” (FM) group uses a purely forward predictive strategy to solve the motor learning task (i.e., their four cerebellar microcomplexes implementing the inverse corrector models remain “switched off”). The “inverse model” (IM) group employs a purely inverse dynamics corrective strategy to adapt to the unknown angular bias. The “coupling model” (CM) group uses the full motor control architecture based on active and adaptive inverse and forward models. We perform intergroup quantitative comparisons of rotation adaptation performances, and compare our simulation results to the experimental data with humans reported by Huber et al. (2004).

#### 2.2.2. Offline consolidation of procedural learning

In the second phase of the protocol, we test to what extent offline sensorimotor processing can enhance the adaptation performance following online training. We consider two groups (CM_sleep_ and CM_wake_) of ten simulated subjects each. Both groups undergo 12 online training trials as described before. Then, a probe test is performed (trial 13) to evaluate the extent of online rotation adaptation in both groups (Figure 2B). Subsequently, simulated subjects from group CM_sleep_ undergo an offline learning process consisting of a set of 48 trials (4320 trajectories randomly replayed) during which no sensory feedback is provided to the system. As previously mentioned, during offline processing the teaching signal can only be computed based on the predictions provided by the forward models. The inverse corrector model is only allowed to adapt its dynamics when teaching information is available. The CM_wake_ group does not undergo the offline processing/consolidation phase. Finally, we perform a second probe test (trial 14) to compare the performance of the two simulated groups (i.e., with *vs.* without offline consolidation).

#### 2.2.3. Data analysis

Two measures assess the motor learning performance, namely the directional and distance errors. The directional error is the angle between the ideal straight trajectory to the target (dotted green line in Figure 2A) and the line representing the movement at the peak outward velocity (solid black line in Figure 2A; the red line is the actual hand goal-directed trajectory). The distance error is calculated as the Euclidean distance between the target position (*T*) and the final actual position of the hand (*E*). Both errors are normalised and computed independently. We assume that a trajectory is optimal when both errors are minimised, and we quantify the impact of offline procedural learning as the percentage performance change between trial 13 and 14. The percentage of performance change is defined as the percentage of error decrease between trial 13 and trial 14 ($\frac{\text{error}(\text{trial}14)-\text{error}(\text{trial}13)}{\text{error}(\text{trial}13)}*100$, as is Huber et al., 2004). We assess performance change for both directional and distance errors.

We compute the statistical significance of intergroup performance differences by means of an ANOVA analysis (with *P* < 0.01 considered as significant).

## 3. Results

Prior to the execution of the main series of simulations (see below), we verified that learning in forward predictor cerebellar models would result in accurate estimates of future sensory outcomes of motor commands, and that adaptation in inverse correctors could significantly improve the accuracy of motor command execution (see Supplementary Figure S4). Furthermore, we investigated the neural information processing and connectivity properties subserving effective information processing and learning capabilities in the simulated control system (see Supplementary Results).

### 3.1. Coupling forward and inverse models enhances online sensorimotor adaptation

**Figure 3:**
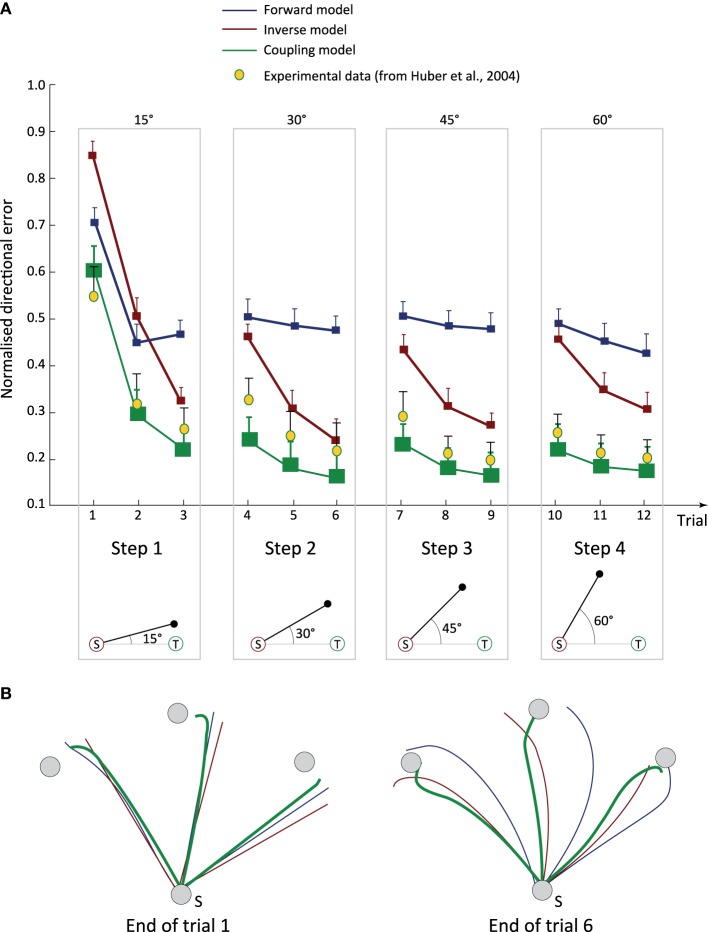
**Coupling forward and inverse models enhances online sensorimotor adaptation. (A)** Time course of directional error through training. The mean normalised directional error (averaged over all subjects) is plotted as a function of training trials. Subjects from the *FM* group (blue line) exhibited fast adaptation capabilities but their performance reached a plateau after a few trials. Subjects from the *IM* group (red lines) showed a slower learning capabilities compared to IMs, but achieved a higher level of performance once adapted to the task. Subjects using a coupled cerebellar model (i.e., *CM* group, plain green curves) showed the best adaptation performance, and were the only ones able to fit human experimental data (yellow dots). **(B)** Examples of three target-directed trajectories at two training steps. The blue dotted (resp. red dashed) lines indicate a sample solution found by a purely forward model, FM, (resp. inverse model, IM) subject. The green solid lines denote the trajectories obtained by the coupling scheme model, i.e., CM subjects.

We tested three groups of subjects, namely “forward model” (FM), “inverse model” (IM), and “coupling model” (CM) group, on the rotation adaptation task (Figure 2B). All three groups learnt to solve the task and to cope with the progressively larger unknown angular bias (see Figure 3A for their performance in terms of directional error and Figure 3B for a representative set of trajectories at two different stages of training). Simulated FM subjects (blue curves) adapted rapidly, but they reached a plateau after trial 2 with no further minimisation of the angular error until the end of the training process (trial 12). Interestingly, the transitions between steps (i.e., with an increasing bias at trials 4, 7, and 10) did not have any significant impact on the performance of the FM group, reflecting fast motor learning capabilities. Adaptation was slightly slower in IM subjects (compared to the FM group), but they succeeded in minimising the directional error within each training session (red curves in Figure 3A), going significantly beyond the performance of FM subjects (ANOVA, *F*_(1, 18)_ = 481.3, *P* < 0.001). The learning profile of IM subjects was rather stereotyped during steps 2, 3, and 4. After each step transition, the IM group performance was impaired by the larger unknown angular bias, with a consequent increase of the directional error.

CM subjects using the coupled cerebellar internal models performed significantly better than both IM and FM groups over the entire training (Figure 3A, green curves ANOVA, *F*_(1, 18)_ = 1103.43, *P* < 0.001 and ANOVA, *F*_(1, 18)_ = 3761.15, *P* < 0.001 for IM and FM groups, respectively). CM subjects showed faster sensorimotor adaptability and higher accuracy over time. Although their performance slightly diminished after each step transition, the directional error decreased significantly in the next trial (ANOVA, *F*_(1, 18)_ = 1102.76, *P* < 0.001). Notably, simulated CM subjects exhibited the only comparable performance (qualitatively) to those observed in human subjects solving the same task (Huber et al., 2004) (Figure 3A, yellow data points). By contrast, neither purely forward predictors (FMs) nor purely inverse correctors (IMs) could reach the motor adaptation performance of human subjects.

Similar results held for the distance error of simulated subjects solving the rotation adaptation task (Supplementary Figure S5), with the group employing coupled internal models demonstrating significantly larger adaptation capabilities. As for the directional error scenario, FMs minimised the distance error better than IMs, particularly for small values of the angular bias (i.e., for 15° and 30°; Figure S5).

### 3.2. Coupling forward and inverse models supports offline sensorimotor consolidation

We investigated the possible performance enhancement induced by the offline procedural consolidation by testing two other simulated groups (CM_sleep_ and CM_wake_) on the rotation adaptation task (see section 2). We recall that both groups underwent the entire online training (12 trials) plus the first probe test (trial 13; Figure 2B). Subsequently, only the CM_sleep_ group had access to an offline learning period. We then compared the adaptation performance of these two groups on a second probe test (trial 14).

#### 3.2.1. Performance improvement following offline sensorimotor consolidation

**Figure 4:**
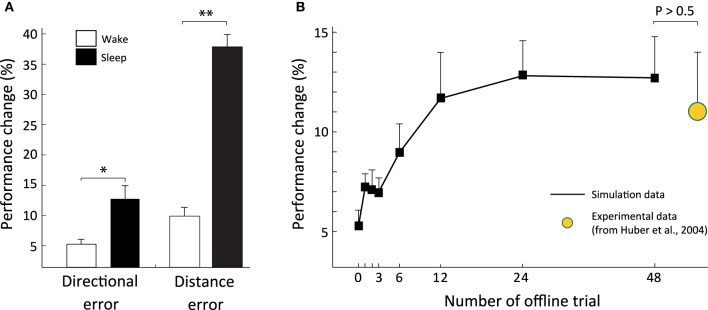
**Coupling forward and inverse models supports offline sensorimotor consolidation. (A)** Significant performance improvement (in terms of both directional and distance errors) after offline consolidation (^*^P < 0.01, ^**^P < 0.001). **(B)** Performance improvement as a function of the duration (number of replayed trials) of the offline consolidation period. Human experimental results from Huber et al. (2004) are shown in yellow.

CM_sleep_ and CM_wake_ subjects had similar performances during the first probe test on trial 13 (ANOVA, *F*_(1, 18)_ = 0.33, *P* > 0.5; *F*_(1, 18)_ = 0.01, *P* > 0.5 for angular and distance error, respectively; not shown). By contrast, during the second probe test on trial 14, CM_sleep_ subjects exhibited a significantly larger performance enhancement than CM_wake_ subjects (Figure 4A). Concerning the directional error, CM_sleep_ subjects had a performance improvement of 12.7±2.1% (mean ± s.e) compared to the probe test on trial 13, i.e., after their offline consolidation period. This enhancement was significantly larger than the control group CM_wake_ (ANOVA, *F*_(1, 18)_ = 13.01, *P* < 0.01), which exhibited a performance improvement of 5.2±1% relative to trial 13. For the distance error, we observed an even larger performance enhancement in CM_sleep_ subjects. With respect to trial 13, they improved their performance by 38±2%, which was significantly larger than CM_wake_ subjects (ANOVA, *F*_(1, 18)_ = 122.77, *P* < 0.001), showing a progression of 9.8±1.5%. note that the performance improvement of the control population CM_wake_ (i.e., the group that did not undergo offline consolidation) reflects the sensorimotor learning during the second probe test on trial 14.

#### 3.2.2. Time course of the offline consolidation process

To address how the performance improvements of CM_sleep_ subjects varied as a function of the duration of the offline consolidation period, we ran a new series of simulations with 8 different CM_sleep_ groups (10 individuals each). All groups underwent the entire online training (12 trials) and the first probe test (trial 13; Figure 2B). As expected, the 8 groups showed equivalent performance on the rotation adaptation task when tested on probe trial 13 (ANOVA, *F*_(1, 78)_ = 0.59, *P* > 0.5; *F*_(1, 78)_ = 0.00072, *P* > 0.5 for angular and distance error, respectively, not shown). Then, each group had access to an offline learning period of different length (1, 2, 3, 4, 6, 12, 24, 48 trials, respectively), and was tested on the second probe test (trial 14) afterwards. The performance gain induced by the offline consolidation increased significantly with “sleep” duration (repeated-ANOVA, *F*_(7, 63)_ = 22.05; *P* < 0.001; see Figure 4B for the directional error). However, the improvement reached a plateau after 12–24 duration trials with no significant increase for longer offline periods (ANOVA, *F*_(1, 18)_ = 0.02, *P* > 0.5). The plateau value of the offline-dependent performance gain (e.g., on trial 48) was comparable to that observed experimentally on human subjects after one night of sleep (Huber et al., 2004) (11±3%, yellow data in Figure 4B). A similar time course was observed for the performance enhancement concerning the distance error, with a plateau-like effect starting after 12–24 trials of offline learning (not shown). Notably, both for directional and distance errors, we did not observe any fall in performance enhancement during offline overtraining (i.e., for a number of offline trials >50).

## 4. Discussion

This study addresses the functional role of coupling internal cerebellar models in both online and offline sensorimotor adaptation. The proposed model takes inspiration from the cerebellar microcomplex circuit. We adopted a composite system based on the coupling of a feed-forward architecture (Kawato et al., 1987) *and* a recurrent architecture (Dean et al., 2010). In the model, motor control learning depends on long-term synaptic plasticity mechanims (both potentiation, LTP, and depression, LTD) that adapt the input–output dynamics of the simulated cerebellar circuit to contextual changes. The cerebellar microcomplex architecture implements both forward predictor and inverse corrector models. We assess their relative and complementary contributions to motor learning by simulating the rotation adaptation task used by Huber et al. (2004) to study procedural learning in humans.

Our main results suggest that the coupling of internal models enhances online adaptation performance and thus allowing the simulated control system to reach the online adaptation capabilities of humans. We observed that the forward predictive model and the inverse corrective model lead to complementary corrections and that coupling those internal models yielded significant performance improvements.

Furthermore, we provide a mechanistic interpretation of the procedural performance improvement observed in humans after one night of sleep following online training (Huber et al., 2004). The model also predicts that the coupling of forward and inverse internal models constitutes a necessary condition for the offline consolidation of procedural sensorimotor memories.

### 4.1. The internal model hypothesis

Experimental evidence suggests that the cerebellum can acquire and store internal forward and inverse models (Wolpert et al., 1998; Kawato et al., 2003; Ito, 2005; Pasalar et al., 2006). Further evidence indicates that forward models may be implemented in the spinocerebellum-magnocellular red nucleus system and inverse models in the cerebrocerebellum-parvocellular red nucleus system (Kawato et al., 1987). Internal cerebellar models are thought to influence a large body of sensorimotor tasks, such as motor learning and control (Ito, 1984; Jordan and Rumelhart, 1992; Miall et al., 1993; Wolpert and Miall, 1996; Mulliken et al., 2008; Shadmehr and Krakauer, 2008), state and sensory estimations (Goodwin, 1984; Miall et al., 1993; Miall, 1998), sensory cancelation (Bell et al., 1997), and context predictions (Wolpert and Kawato, 1998; Eskandar and Assad, 1999). Additionally, some neurocomputational and robotic studies have validated the viability of systems implementing internal models to solve complex motor learning tasks (Carrillo et al., 2008; Laschi et al., 2008; Saegusa et al., 2009). Nevertheless, the coupling of internal models remains only partially investigated. To the best of our knowledge, the first study postulating that forward models may generate sensory error signals driving adaptation in inverse models has been presented by Jordan and Rumelhart (1992). The authors demonstrated that certain classical motor learning problems could be solved by using paired internal models as the main component of a larger adaptive system. Wolpert and Kawato (1998) proposed an architecture based on multiple coupled inverse and forward models forming functional units. The authors showed that a large repertoire of behaviours can be generated with a limited number of paired models.

Compared to these earlier works, we propose a biologically inspired cerebellar model, thus allowing us to characterise some relevant properties of the biological counterparts (see Supplementary Results). With respect to the model by Wolpert and Kawato (1998), the forward predictors implemented in our cerebellar architecture do not serve to choose the appropriate inverse models for a given context. Instead, they participate in both training inverse corrective models and influencing the motor output by sending future state predictions to a high-level (cortical) controller. In our study, the task is limited to a single tool manipulation, and aims at understanding how forward predictive and inverse corrective models could work cooperatively to improve motor performance in an isolated task. Moreover, this study considers the possible implications of internal model coupling in offline consolidation of procedural learning (occurring during sleep), which was not addressed in previous investigations.

### 4.2. Motivations for a spiking implementation

Both computational and biological reasons justify the choice of a spiking implementation in this study. First, we were interested in the fast dynamics of different neural populations in the cerebellar microcircuitry, thus requiring the action potential to be modelled. For instance, we investigated how the population of granular cells could re-encode the afferent MF signals into a sparse representation, both in time and space (this section is described in Supplementary Results). Furthermore, a spiking implementation gave us the opportunity to investigate to what extent the hetero-synaptic long term depression at the level of the parallel fibre to Purkinje cell synapses (as described by Ito and Kano, 1982) could induce the formation of sensorimotor memories. Finally, a spiking implementation offers a level of detail that helps to better understand how the cerebellum processes information. The current model can easily be extended to provide more accurate details of neural units, connectivity properties, and plasticity mechanisms. These may increase the performance of the system by maximising information processing, increasing memory storage, and/or optimising adaptation speed and accuracy.

### 4.3. Motivations for the choice of the behavioural task

The simulated cerebellar microcomplex is used to adapt the dynamics of a fairly simple control system consisting of a two degrees of freedom arm moving in a 2 dimensional space. We have modelled a very restricted portion of the cerebellar cortex to achieve this procedural adaptation. Furthermore, to implement the simulated version of the rotation adaptation task, we have assumed a few simplifications that allowed us to model the paradigm using a torque controller. In the experimental setup used by Huber et al. (2004), a cursor represents the position of the hand, and a subject must extract the error by comparing the expected position of the cursor and the real position of the cursor (given by the visual feedback). In comparison, in the simulated version of the task, we inject the angular bias before the generation of the force (in the trajectory generator module); as a consequence, the trajectory of the simulated arm is deviated with the same angular bias. Then, in our framework, we provide the system with the estimation of the joints to calculate the error that will drive adaptation (we do not model the visual system). In this simplified scenario, the controller must adapt the torque value to cancel the deviation. Consequently, the simulated version of the task does not strictly emulate the experimental one; but since in the latter case, the conflict between the expected and sensed visual sensory feedback ultimately results in an adaptation of the torque, this simplification seems reasonable.

There are a few reasons why we use the rotation adaptation task for this theoretical work. First, this task can easily be reproduced in a simulated environment. Second, solving the task requires the formation of procedural memories where both predictive and corrective signals can change the rate of adaptation. Finally, the rotation adaptation task has previously been used to study the influence of sleep on procedural learning, and the performance of human subjects was quantitatively measured both during online adaptation and after offline consolidation (Huber et al., 2004).

The model presented in this paper can successfully solve the rotation adaptation task with only a limited number of units. However, in order to adapt the dynamics of more complex systems in a various set of behaviours, the system would need a greater extent of neuronal resources. To achieve good performance with limited resources, one possible solution may be to use a modular approach as previously proposed by Wolpert et al. (1998). The coupling model as proposed in this paper would then be taken as a functional unit, and combining the output of several units could generate various behaviours. Because one unit could be used in different contexts, a large repertoire of behaviours could be generated, even with a limited number of modules.

### 4.4. Training inverse corrective models

The teaching signal used to train an inverse model relies on the difference between the actual and correct motor command. Yet, an autonomous agent must extract this motor error using information encoded in sensory coordinates (the difference between the desired state and the perceived state). This is known as the distal error problem (Jordan and Rumelhart, 1992) and has been addressed in other studies by using different approaches such as direct inverse modeling (Widrow and Stearns, 1985), distal supervised learning (Jordan and Rumelhart, 1992) or Feedback-Error-Learning scheme (Gomi and Kawato, 1993). In our study, we do not solve the distal error problem and assume that the transformation from sensory error to motor error is already known by the system and then conveyed to the inverse corrective model as a teaching signal.

During online learning, we apply the sensory to motor error transformation on the difference between the desired state and the delayed sensory feedback. During offline learning, since the latter information is not available, we apply the transformation on the difference between the desired state and the predicted sensory feedback. In both cases, the output of the transformation is then used as a supervised learning signal to train the inverse corrective model. We use a simple (yet non-biological) switch to rewire the teaching signal used to train the inverse model when performing online learning versus offline consolidation. Such a re-wiring is highly unlikely to occur in a biological system. A more elegant solution would be to have an additional system that integrates both sensory feedback and feedback prediction into a coherent representation of the state of each joint (for example by implementing a Bayesian integrator such as a Kalman filter). The parietal cortex might subserve such a function (Desmurget et al., 1999; Grea et al., 2002; Shadmehr and Krakauer, 2008). The difference between the desired state of the joints and the internal representation could then be used to train the inverse model (after applying the transformation) with the same principle both during online learning and offline consolidation. During offline consolidation, in contrast with online learning, only the predicted sensory feedback can be used to determine the state of each joint. The internal representation would therefore be degraded if the forward model were not fully and accurately formed.

### 4.5. Cerebellar information processing and connectivity properties

The modelled cerebellar microcomplex is highly simplified with respect to its biological counterpart. The model neglects the function of cerebellar interneurons (Golgi, stellate and basket cells), which are thought to play a role in processing timing information (Desmond and Moore, 1988; Fiala et al., 1996; Kistler et al., 2000; Yamazaki and Tanaka, 2007), enhancing the signal-to-noise ratio (Hirano et al., 2002; Garrido et al., 2007), and providing the biological resources for the implementation of covariance-based rules [see Sejnowski (1977); Dean et al. (2010) for reviews]. Furthermore, the adaptation has been modelled by long term synaptic plastic changes between parallel fibres and Purkinje cells (a heterosynaptic LTD and homosynaptic LTP), and all other connection weights were fixed at the creation of the network. Many other plasticity mechanisms have been reported in the cerebellum, and it is likely that every connection undergoes plasticity mechanisms [see for example Hansel et al. (2001); Zhang and Linden (2006); Schonewille et al. (2010), and Schonewille et al. (2011)]. However, given that (1) the functions of these plasticity mechanisms and variety of neural types are still poorly understood, and that (2) this was not the main focus of this study, the model does not account for these properties. A further development of this work should take into consideration these processes to address their possible functions and consequences in the coupling scheme. For example, one extension of this model should include synaptic plasticity between the mossy fibres and deep cerebellar nuclei as described by Pugh and Raman (2006); Zhang and Linden (2006). This synaptic plasticity is thought to be triggered by an activity rebound of the DCN, following the high hyperpolarisation level of the cell (Pugh and Raman, 2006). Yet, functional implications are still unclear: memory may be acquired first in the cerebellar cortex and then be transferred to the cerebellar nuclei. This synaptic plasticity would therefore contribute to the persistence of memories (Masuda and Amari, 2008). Alternatively, the cerebellar cortex could store timing-related information whereas the deep cerebellar nucleus would be more important for storing the amplitude of the response (Medina and Mauk, 1999).

Nevertheless, our simulations allowed us to investigate some neural information processing and connectivity features at the cerebellar level, which are likely to be relevant to sensorimotor adaptation and offline procedural consolidation (see Supplementary Results).

In our model, the simulated population of granule cells provides a sparse representation (Willmore and Tolhurst, 2001; Assisi et al., 2007) of mossy fibre inputs (see Supplementary Results). This result is in line with experimental and theoretical findings suggesting that a sparse code in the population of granule cells should minimise interferences across learning tasks, optimize neuronal resources by reducing redundancy, and facilitate downstream signal integration at the level of Purkinje cells (Miyashita, 1988; Schweighofer et al., 2001; Brunel et al., 2004; Olshausen and Field, 2004; Philipona and Coenen, 2004).

We also observe that a bistable behaviour of the forward model (i.e., with active output only in the presence of accurate predictions) can be relevant to both online and offline processing (see Supplementary Results). Indeed, during online processing, if forward models were providing inaccurate predictions, then the trajectory planning would lead to catastrophic performance. During offline processing, since forward predictions were used to compute the teaching signal to drive adaptation in the inverse corrector models, suboptimal forward estimates could lead to destructive offline learning. There exists experimental evidence suggesting that the accuracy of a forward model is maintained through adaptive processes driven by sensory prediction errors (Mazzoni and Krakauer, 2006; Miall et al., 2007), but little is known concerning the first step of learning of a forward model. We propose that a bistable behaviour of the forward model might reflect the accuracy of the prediction.

We also suggest that when the teaching signal that drives learning in cerebellar internal models is unavailable or nil, multiple long term modifications at the level of cerebellar synapses must compensate for each other, assuring the maintenance of the stored sensorimotor associations. The absence of a teaching signal can occur in multiple scenarios: (1) during offline consolidation when a forward model is unable to predict the sensory consequences of a motor command; (2) during online adaptation, when the sensory consequences of a movement are absent or incomplete; and (3) after adaptation when there is nothing left to be learnt. In our model, two asymmetrical processes at the level of the PF–PC synapse mediate adaptation. First, a homosynaptic LTP is driven by the activity of the granular layer and occurs before knowing whether or not a teaching signal would be available. Second, the activity of the climbing fibre acts as a teaching signal, and, if present, depresses the activity of the previously activated and reinforced PF–PC synapses. Hence, in the absence of a teaching signal, homosynaptic LTP and heterosynpatic LTD must precisely compensate each other to avoid disturbing the learnt sensorimotor association. This homeostatic property was of primary importance to endow our simulated system with offline learning capability (if the forward models were unable to predict the sensory consequences of a motor command during offline processing, then the previously learnt sensorimotor associations should persist unchanged). We observed that LTP–LTD compensation may also depend on the PC to DCN synaptic convergence (see Supplementary Results). In our simulations, LTP–LTD compensation occurs when the number of PCs projecting to each DCN is greater than 20. Palkovits et al. (1977) showed that the majority of cerebellar Purkinje cells synapse onto neurons in the DCN; 90% of DCN neurons are contacted by Purkinje cell terminals, with an estimated synaptic convergence of 26:1 (Palkovits et al., 1977).

### 4.6. Consolidation of procedural memories

A substantial number of experimental works have confirmed the beneficial effect of sleep on procedural memories (Plihal and Born, 1997; Stickgold et al., 2000b; Fischer et al., 2002; Gais et al., 2002; Mednick et al., 2003; Walker et al., 2003; Korman et al., 2007). Although memory consolidation is often considered as a stabilisation phase, there are many examples of procedural tasks in which the performance of subjects retested after a night of sleep improved significantly with respect to the last evening session (e.g., Huber et al., 2004; Walker and Stickgold, 2004). Our study postulates that the coupling of internal models might be relevant to this sleep-dependent procedural consolidation process.

As previously mentioned, offline learning in our model relies on the assumption that the neural patterns corresponding to sequences of actions carried out during online training can be replayed offline. Several studies suggested that the firing patterns observed in the neuronal ensembles of rodents are reactivated during non-rapid eye movement (NREM) sleep. These reactivations were mainly observed in hippocampal place cells after the realisation of a spatial task (Wilson and McNaughton, 1994; Kudrimoti et al., 1999). Spike sequences have also been shown to be repeated in hippocampal cells during NREM sleep (Nadasdy et al., 1999), and the temporal order of place cells firing conserved during repetitive moves (Lee and Wilson, 2002). This offline replay of hippocampal activity has been proposed to be involved in the consolidation of newly encoded spatial information. Several neuroimaging studies in humans have explored the possibility that patterns of brain activities elicited during initial training could be replayed during subsequent sleep. Using PET imaging, Maquet et al. (2000) have shown that activation patterns elicited during practice of a serial-reaction-time motor skill task prior to sleep reappeared during subsequent REM sleep episodes, whereas this activity was not present in control subjects who did not receive day-time training.

Significant sleep-dependent memory benefits were observed after 8 h of night sleep, but also after shorter naps of 1 or 2 h (Mednick et al., 2003; Korman et al., 2007; Nishida and Walker, 2007). Expectedly, longer sleep durations led to greater performance improvements (Stickgold et al., 2000a; Walker et al., 2003), but the “optimal” duration of sleep remains unclear (Diekelmann and Born, 2010). Our model allowed us to study the time course of the offline consolidation process, and we also found that the performance enhancement increased with the duration of “simulated sleep” periods. Consistently with experimental findings, we showed that benefits could already be observed after short offline consolidation periods (e.g., 6 trials), but that longer durations (i.e., 48 trials replayed offline) would lead to significantly larger improvements in motor adaptation. Interestingly, our simulations showed the occurrence of a plateau in performance enhancement after a certain duration of offline consolidation, suggesting that longer simulated sleep periods would not further improve procedural adaptation. The “optimal” duration of the offline consolidation process may depend, among others, on the complexity of the motor learning task and the level of performance achieved before each sleep period. The coupling model presented here may help to characterise some parameters determining the appropriate duration of offline consolidation. For instance, employing the model in different procedural tasks might provide some hints on possible correlations between task complexity and optimal offline consolidation duration. The model could also be used to study the influence of the quality of the encoded information in internal models on the offline consolidation process. For example, we might quantify to what extent the accuracy of forward predictions might modulate the time-to-plateau or the shape of the offline optimisation time course.

The study presented here suggests a potential role of the cerebellum in consolidating procedural memories after an initial training. To the best of our knowledge, only a few experimental works have addressed this issue. Maquet et al. (2003) trained two groups of human subjects (one control and one sleep-deprived) on a smooth pursuit task. In both groups, authors observed an increase in brain responses to a learnt trajectory as compared to an experienced trajectory. However, in a probe test on the following day, the authors observed behavioural and functional intergroup differences. Compared to the sleep-deprived group, the performance of control subjects was improved and an increased functional connectivity was observed between the superior temporal sulcus and the cerebellum. These differences were interpreted as sleep-related plastic changes during motor skill learning in areas involved in smooth pursuit eye movements, such as the cerebellum (Maquet et al., 2003). In another study by Walker et al. (2005), fMRI data showed an interesting change in the representation of a motor memory after a night of sleep. Following sleep relative to wake, regions of increased activation were expressed in the left cerebellum. Also, the right primary motor cortex, the medial prefrontal lobe, and the hippocampus showed augmented activity, thus suggesting that post-training consolidation engaged the modification of a complex network involving the interactions of multiple structures (Walker et al., 2005). Recently, it has been suggested that the cerebellum might be involved in sleep-dependent offline consolidation in tasks related to the optimisation of timing (Siengsukon and Boyd, 2009). Lewis et al. (2011) examined post-sleep improvement in task requiring motor and perceptual timing. They observed that the cerebellum was likely to be involved in the consolidation of motor timing and showed that the brain state during retention (sleep or wake) modulated subsequent responses in the lateral cerebellum (but also the striatum and the supplementary motor area). However, these studies could not describe the cerebellar computation involved in offline sensorimotor consolidation. Further experiments specifically designed to study the underlying cerebellar mechanisms mediating offline consolidation of procedural memories shall be carried out. The modelling approach presented here can complement these investigations by providing a mechanistic view of how the cerebellum may be involved in offline sensorimotor consolidation.

### Conflict of interest statement

The authors declare that the research was conducted in the absence of any commercial or financial relationships that could be construed as a potential conflict of interest.
