# Supplementary material for: Coupling internal cerebellar models enhances online adaptation and supports offline consolidation in sensorimotor tasks
Source: Front Comput Neurosci. 2013 Jul 15;7:95. doi: 10.3389/fncom.2013.00095 (PMC3711060; doi:10.3389/fncom.2013.00095)
Supplement: Supplementary file 1 [file 31806_Arleo_Presentation1.PDF]

Coupling internal cerebellar models enhances online adaptation and supports offline consolidation in sensorimotor tasks

Jean-Baptiste Passot[1], Niceto R. Luque[2], Angelo Arleo[1,*]

[1]CNRS – UPMC Univ Paris 6, Unit of Neurobiology of Adaptive Processes, UMR 7102, F75005, Paris, France

[2]UGR – Computer Architecture and Technology Department, University of Granada, 18071 Granada, Spain

*Corresponding author, email: angelo.arleo@upmc.fr, phone / fax: +33 (0)1 44.27.27.80 / .26.69

Supplementary Methods

Cerebellar microcomplex model

S1.1 Circuit model

The cerebellar microcomplex model (Fig. 2 B in the main text) captures the main processing stages and connectivity layout (e.g. convergence/divergence ratios) of its biological counterpart (see Ito, 2006 for a recent review). model, the mossy fibre (MF) layer consists of 200 cell axons. MFs activate a population of 10^4 granule cells (GCs), which produce a sparse representation of the input state space (see in the main text, Sec. 3.1). Each MF is connected to a GC with a probability $P_{MF-GC} = 0.02$. Thus, each GC receives an average of 4 MF inputs and each MF projects onto approximately 200 GCs. GCs project onto a population of 100 Purkinje cells (PCs) through the parallel fibres (PFs), i.e. GCs' axons. Each GC can create a connection to a PC with a probability $P_{GC-PC} = 0.75$. Thus, each PC can receive about 7500 input connections from PFs. MFs also excite a population of 50 deep cerebellar nuclei (DCN) cells by all-to-all connections ($P_{MF-DCN} = 1$). In addition to the excitatory MF input, the discharge of each unit of the DCN layer —which constitutes the output of the cerebellar network— is modulated by the inhibitory action of 2 afferent PCs. This simplification of the model (a real DCN is targeted by tenths to hundreds of PCs, Palkovits et al., 1977) has been adopted to merely bypass a computational load issue: given that each simulated PC integrates the signal of thousands GCs, we could only implement a small number of PCs, and consequently limit the convergence ratio from PCs to DCN. A bidirectional long-term plasticity rule modifies the strength of PF–PC synapses, changing the input-output relationship of the circuit. The teaching signal for the learning rule is conveyed to the PCs by a population of 2000 inferior olive (IO) neurons, whose axons (i.e. climbing fibres, CFs) project onto PCs according to a twenty-to-one connection scheme. The biological IO-to-PCs convergence ratio is actually significantly lower, but we used this connectivity layout to functionally compensate for the effect of the aforementioned low synaptic convergence from PCs to DCN in our model (Fig. A1). Finally, the highly simplified microcomplex model does not account for cerebellar interneurons, which are likely to be involved in denoising neurotransmission in the cerebellum (Hirano et al., 2002), conveying timing information (Desmond and Moore, 1988; Yamazaki and Tanaka, 2007; D'Angelo and De Zeeuw, 2009), and providing the biological substrate for the implementation of covariance-based learning rules (Sejnowski, 1977; Dean et al., 2010). Parameters of a simulated cerebellar microcomplex are summarized in table A1

Different instances of the microcomplex circuit are used to implement 4 inverse corrector models (IMs) and 4 forward predictor models (FMs). Both IMs and FMs receive as inputs the motor command efferent copies (i.e. the pair of actual torque values $\tau_s'(t)$ and $\tau_s'(t)$), the current state (i.e. the perceived

Unit name	Number of units	Afferent connection	Efferent connection	Connectivity type
MF	200		GR (±200)<br>DCN (50)	Random ($p = 0.04$)<br>All to all
GR	10.000	MF (±4)	 PC (±75)	Random<br>Random ($p = 0.75$)
PC	100	GR (±7500)<br>IO (20)	 <br>DCN (2)	Random<br>Topographic<br>Topographic
IO	2000		PC (1)	Topographic
DCN	50	PC (2)<br>MF (100)		Topographic<br>All to all

Table A1. Number of units and connectivity between layers for a simulated microcomplex

angular position $\theta_s(t)$ and $\theta_e(t)$ of shoulder and elbow joints, respectively) and the desired *next* angular positions θ_s, θ_e and velocities $\dot{\theta}_s, \dot{\theta}_e$ for both joints (shoulder s and elbow e). IMs learn to map desired states (positions and velocities) into torque corrections to compensate for local errors and deviations during the reaching movement. IM outputs are torque correction values $\hat{\tau}_s, \hat{\tau}_e$ for the shoulder and elbow joint, respectively. Among the 4 IMs, 2 learn to correct errors in the positive and negative ranges of the shoulder torque, $\hat{\tau}_s^+(t)$ and $\hat{\tau}_s^-(t)$, respectively. The other 2 learn to correct errors in the positive and negative ranges of the elbow torque, $\hat{\tau}_e^+(t)$ and $\hat{\tau}_e^-(t)$, respectively. The final motor commands sent to each arm joint are then $\tau_s' = \tau_s + \hat{\tau}_s$, and $\tau_e' = \tau_e + \hat{\tau}_e$, with τ_s, τ_e denoting the ideal torque values computed by the high-level controller (see main text, Fig. 1A).

FMs learn to infer the next shoulder and elbow positions and velocities based on the current state and torque commands applied to each joint. The FM outputs at time t are the predicted angular positions $\hat{\theta}_s(t)$ and $\hat{\theta}_e(t)$, the predicted angular speeds $\hat{\dot{\theta}}_s(t)$ and $\hat{\dot{\theta}}_e(t)$. Predictions are occurring 100 ms before the actual sensory feedback for a given context.

S1.2 Neuronal models

We model each MF as the axon of a leaky integrate-and-fire neuron whose membrane potential $V(t)$ dynamics is defined as:

$$C \frac{dV(t)}{dt} = g_{leak}\Big(V_{leak} - V(t)\Big) + I(t) \tag{A1}$$

where C denotes the membrane capacitance and g_{leak} the leak membrane conductance —i.e. $\tau = C/g_{leak}$ is the membrane time constant; V_{leak} is the resting membrane potential, and $I(t)$ the total synaptic drive. Whenever the membrane potential reaches a threshold V_{th} the neuron emits a spike. We used a time step $dt = 1$ ms for numerical integration.

GCs, PCs and DCN neurons are conductance based leaky integrate-and-fire units (similar to Carrillo

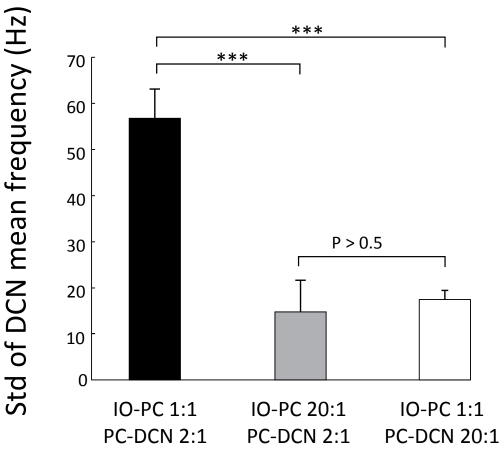

Figure A1. Stabilisation of DCN firing rate under different connectivity layouts. IO neurons have a very low discharge rate ($\sim$ 1 to 2 Hz when recorded in vivo) (Gibson et al., 2004). In a network model with only 2 PCs targeting each DCN cell, and with biologically-plausible 1:1 IO-to-PC connections, the resulting DCN activity is rather unstable ($CV_{DCN} = 0.57 \pm 0.06$). To compensate for this effect, each PC of the model receives 20 IO projections, which leads to a more stable DCN activity ($CV_{DCN} = 0.16 \pm 0.08$), with a mean frequency comparable to that of a cerebellar network model in which the IO-to-PC connection ratio is 1:1 and the PC-to-DCN connection ratio is 20:1 (which is more biologically plausible). This homoeostatic condition indicates that, in the presence of a nil error signal conveyed by the IO, LTD and LTP at PF–PC synapses can compensate each other on a long time scale basis, preventing DCN activity from diverging.

et al., 2008) described by the following equation:

$$C\,\frac{dV(t)}{dt} = g_{leak}(t)\Big(V_{leak} - V(t)\Big) + g_{exc}(t)\Big(V_{exc} - V(t)\Big) + g_{inh}(t)\Big(V_{inh} - V(t)\Big) \quad \text{(A2)}$$

where the membrane potential $V(t)$ depends on an excitatory synaptic conductance g_{exc}, an inhibitory conductance g_{inh}, and a leaky conductance g_{leak}. V_{leak}, V_{exc}, and V_{inh} are the corresponding resting potentials. Again, when $V(t)$ reaches a threshold V_{th} the cell emits a spike. All active conductances $g_{leak}(t)$, $g_{exc}(t)$, $g_{inh}(t)$ vary according to:

$$g(t) = g \sum_j W_j \int_{-\infty}^{t} \exp\Big(\frac{t-t'}{\tau}\Big)\delta(t-t')dt \quad \text{(A3)}$$

where g is the maximal conductance, W_j is the efficacy of the projection from a presynaptic neuron j (with hard bounds $[0,1]$), τ is the synaptic time constant, t' is the time of a presynaptic spike, and $\delta(t-t')$ is a Dirac function equal to 1 only when the presynaptic neuron emits a spike at time t'.

The discharge of each IO neuron is modelled by a discrete Poisson spike-train generator with rate $r(t)$ determined by the corresponding teaching signal (see below).

Table A2 provides the parameter settings for all neuronal models used in our simulations.

Neuronal parameters					
		DCN	GR	PC	MF
V_{th}	mV	-60	-60	-60	-60
C	pF	2	2	2	2
g_{leak}	nS	2	2	2	2
V_{leak}	mV	-70	-70	-70	-70
g_{exc}	nS	0.1	0.2	60	-
V_{exc}	mV	0	0	0	-
g_{inh}	nS	2	-	-	-
V_{inh}	mV	-80	-80	-80	-
τ_{leak}	ms	20	20	20	20
τ_{exc}	ms	0.5	0.5	0.5	-
τ_{inh}	ms	10	-	-	-

Plasticity parameters		
Model	LTD (β)	LTP (α)
Forward	-0.5	1
Inverse	-0.025	0.1

Table A2. Parameter settings for neuronal and plasticity models.

S1.3 Encoding MF cerebellar inputs

MFs constitute the main input stage of the cerebellar microcomplex (Eccles et al., 1967). In the model, they carry information about desired future states (for the input of inverse corrector microcomplexes) and about current states and torque commands (for the input of forward predictor microcomplexes). For each input variable, a population of 50 MFs acts as a family of radial basis functions encoding the values of a variable. More specifically, the input current $I_i(t)$ (Eq. A1) of each MF neuron i is:

$$I_i(t) = \gamma + \exp\Big(- \frac{(x(t) - \mu_i)^2}{2\sigma^2} \Big) \tag{A4}$$

where μ_i and σ^2 are the centre and the variance of the kernel associated to the MF i, respectively. The distribution of all kernel centres μ_i covers the input space uniformly and the variance parameter σ^2 ensures a small overlap between MF responses. The γ constant factor in Eq. A4 endows each MF neuron with intrinsic spontaneous activity at about 5 Hz, whereas the parameters regulating the discharge of MFs (Eq. A1) limit their activity to 50 Hz.

S1.4 Decoding DCN cerebellar outputs

Decoding DCN activity in inverse corrector models

For the 4 inverse corrector models, the decoding of DCN activity must produce motor command adjustments —i.e. positive and negative shoulder torque corrections ($\hat{\tau}_s^+(t)$, $\hat{\tau}_s^-(t)$) and elbow torque corrections ($\hat{\tau}_e^+(t)$, $\hat{\tau}_e^-(t)$). For each of the 4 inverse models, an average decoding scheme maps DCN outputs into torque corrections. For instance, as shown in Figure S3 for the microcomplex correcting positive torque applied to the shoulder, we take:

$$\hat{\tau}_s^+(t) = \frac{A}{\nu_{max}} \cdot \Big\langle \nu_i(t) \Big\rangle_{i \in DCN} \tag{A5}$$

where $\nu_i(t)$ denotes the instantaneous spike frequency of a DCN neuron i, calculated by averaging over a rectangular sliding window of 50 ms; the normalisation term $\nu_{max} = 200$ Hz is the maximum spike frequency of DCN cells; the scaling factor A determines the maximum correction amplitude. Similarly, the output of the microcomplex devoted to negative shoulder torque correction produces a decoded signal $\hat{\tau}_s^-(t)$. Then, the overall correction for the shoulder torque is:

$$\hat{\tau}_s(t) = \hat{\tau}_s^+(t) + \hat{\tau}_s^-(t) \tag{A6}$$

A similar decoding scheme maps the outputs of the two microcomplexes correcting the elbow torque signal into a signal $\hat{\tau}_e(t)$.

Decoding DCN activity in forward predictor models

For the 4 forward predictor models, the decoding of DCN activity at time t must estimate the angular positions ($\hat{\theta}_s$ and $\hat{\theta}_e$) and the angular velocities ($\hat{\dot{\theta}}_s$ and $\hat{\dot{\theta}}_e$) for the shoulder and elbow, respectively, at time $t + \Delta t$. A population decoding scheme computes the predictions based on the DCN activity (see example in Fig. S4):

$$\hat{\theta}(t) \quad = \quad \frac{\sum_i \nu_i(t) \cdot \theta_i}{\sum_i \nu_i(t)} \tag{A7}$$

$$\hat{\dot{\theta}}(t) \quad = \quad \frac{\sum_i \nu_i(t) \cdot \dot{\theta}_i}{\sum_i \nu_i(t)} \tag{A8}$$

where $\nu_i(t)$ indicates the instantaneous spike frequency of a DCN neuron i, θ_i denotes the preferred angular encoded by a DCN neuron i, and $\dot{\theta}_i$ the preferred angular velocity of a DCN neuron i in the corresponding forward models predicting angular position and velocity of the shoulder and elbow joint. Both preferred angular position and speed values are evenly distributed over the output space (Fig. S4).

The prediction of a forward model is considered as valid if the total activity of its DCN layer overcomes a threshold (20 Hz in our simulation), that is $\sum_{i \in DCN} \nu_i(t) > 20$ Hz. Otherwise, we consider the forward model output as being 'silent' (see Sec. S1.7).

S1.5 Synaptic efficacy and plasticity rules

Only the PF–PC synapses of the simulated microcomplex circuit are plastic —i.e. the model does not account for other plasticity sites in the real cerebellar microcomplex (Hansel et al., 2001; Boyden et al., 2004; De Zeeuw and Yeo, 2005; Pugh and Raman, 2009).

The tuning of non-plastic synapses and neuronal parameters is such that the activity of model cerebellar units is compatible with experimental data. The discharge of simulated GCs is consistent with recent *in vivo* recordings suggesting that joint-related movement inputs generate sustained GC activity at ~ 150 Hz and that two or more MF input spikes are necessary to elicit one GC burst of spikes (Chadderton et al., 2004; Jörntell and Ekerot, 2006; Rancz et al., 2007; Arenz et al., 2008). Simple spikes of model PCs occur at a frequency ≤ 150 Hz (Raman and Bean, 1999) when PCs are activated by PFs. Complex spikes of simulated PCs, caused by a single discharge of the afferent CF, correspond to learning triggering events —we do not simulate high frequency components of the bursts. Finally, DCN neurons discharge at mean firing rates of ~ 20 Hz in the presence of active PC inhibitory inputs (Lamont, 2009), whereas their activity upper-bound is 200 Hz otherwise.

Model PF–PC synapses undergo bidirectional long-term plasticity, i.e. both potentiation, LTP, and depression, LTD. We implement LTP at each PF–PC synapse as a non-associative weight increase triggered by each GC spike, consistent with the homosynaptic rule described by Lev-Ram et al. (2002):

$$\Delta W^{+}_{PF-PC}(t) = \alpha \cdot \delta(t - t_{GC}) \tag{A9}$$

where α denotes the gain factor for LTP. The weight is increased only when the presynaptic GC emits a spike at time $t = t_{GC}$.

We implement LTD at PF–PC synapses as an associative weight decrease triggered by a spike from the IO. This is in agreement with the heterosynaptic plasticity mechanism described by Ito and Kano (1982):

$$\Delta W^{-}_{PF-PC}(t) = -\beta \cdot \int_{-\infty}^{t_{IO}} f(t - t_{GC})\, \delta(t - t_{IO})\, dt \tag{A10}$$

where β is a gain factor for LTD, and the temporal kernel function f correlates each IO spike with the past discharge of a GC (Carrillo et al., 2008). In the model, the largest LTD amplitude occurs when the PC receives an IO spike approximately 100 ms after an input spike from a GC (see Fig. A2), consistent with Safo and Regehr (2008).

The total change in synaptic weight in one PF–PC synapse is then:

$$\Delta W_{PF-PC}(t) = \Delta W^{+}_{PF-PC}(t) + \Delta W^{-}_{PF-PC}(t) \tag{A11}$$

Table A2 provides the parameter settings for the implemented plasticity models.

S1.6 Encoding of error/teaching signals

In both inverse corrector and forward predictor models, IO neurons convey error/teaching signals via the CFs that target PCs and mediate LTD at PF–PC synapses.

Teaching signal for the inverse corrector model

For the 4 inverse models, the error signal accounts for the difference between desired and actual torque command. The teaching signal encodes the perceived motor error, which is the discrepancy between the desired position θ_i of joint i and its real position $\bar{\theta}_i$. The error signal, at time t and for joint i, is then

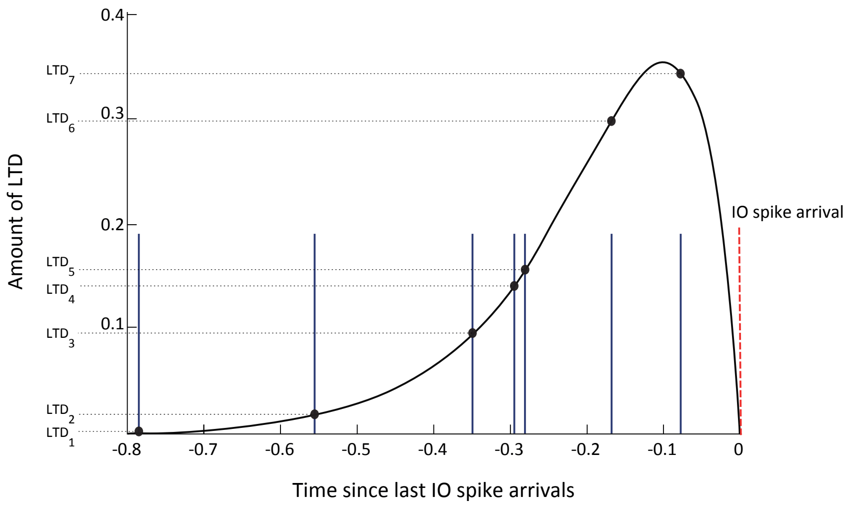

Figure A2. LTD kernel for LTD induction at PF–PC synapses (adapted from Carrillo et al. 2008). The kernel is convolved with the spike train of the afferent PF (all spikes emitted at time $t < 0$). This provides a trace of the past PF activity setting the eligibility of the synapse to depression when the IO neuron (afferent to the PC) emits a spike (at time $t = 0$). In this example the resulting LTD at the PF–PC synapse would be equal to $\sum_{i=1}^{7} LTD_i$.

$\epsilon_i = \theta_i - \hat{\theta}_i$. This error signal is used to modulate the firing rate $r(t)$ of each IO cell. To compute this value, we first extract the positive part of the error signal for joint i, $[\epsilon_i]^+$, which is related to an error in the corresponding agonist muscle, and the negative part $[\epsilon_i]^-$, which is related to an error in the antagonist muscle. For instance, the firing rate $r(t)$ of all IO neurons in the two microcomplexes correcting errors in the positive range ($\hat{\tau}_s^+$) and negative range ($\hat{\tau}_s^-$) of the torque command of the shoulder vary respectively according to:

$$r_{\tau_s^+}(t) = k \cdot \mathcal{H}([\epsilon_s(t)]^+) \qquad \text{(A12)}$$

$$r_{\tau_s^-}(t) = k \cdot \mathcal{H}([\epsilon_s(t)]^-) \qquad \text{(A13)}$$

where $k = 10$ is a scaling factor and $\mathcal{H}$ is the Heaviside function defined such that $\mathcal{H}(0) = 0.1$. According to Eqs. A12, A13 (and see also example in Fig. S3):

- When the cerebellar output equals the output required for the shoulder, i.e. no torque change is needed and $\epsilon_s(t) = 0$, the mean IO firing rates are $r_{\tau_s^+}(t) = r_{\tau_s^-}(t) = 1$ Hz, which make heterosynaptic LTD (Eq. A10) and homosynaptic LTP (Eq. A9) at PF–PC synapses to compensate each other in both microcomplexes —i.e. no adaptation takes place.

- When the cerebellar output is smaller than necessary for the shoulder, i.e. the torque of the shoulder must be increased and $\epsilon_s(t) > 0$, then $r_{\tau_s^+}(t) = 10$ Hz, which makes LTD to take over LTP in the active PF–PC synapses of the corresponding microcomplex. The consequent decrease of PF–PC

synaptic efficacy reduces the inhibitory action of PCs onto DCN neurons the next time that the microcomplex receives the same contextual input —which activates the same PF–PC synapses and then the same PC responses. As a consequence, the population activity of DCN neurons increases, which reinforces the correction signal $\hat{\tau}_s^+(t)$ (according to Eq. A5). In addition, for $\epsilon_s(t) > 0$, the mean IO firing rates $r_{\tau_s^-}(t) = 0$ Hz, which blocks LTD in the active PF–PC synapses of the corresponding microcomplex. Thus, LTP increases and strengthens future inhibitory actions of PCs onto DCN neurons in the presence of the same contextual input to the microcomplex. Then, the correction signal $\hat{\tau}_s^-(t)$ decreases over time. As a consequence, the resultant correction $\hat{\tau}_s(t)$ tends to increase and become positive over training.

- Conversely, when the cerebellar output overshoots the output required for the shoulder —i.e. the torque command of the shoulder must be decreased and $\epsilon_s(t) < 0$— the overall correction $\hat{\tau}_s(t)$ tends to decrease and become negative over training.

During offline consolidation, the sensory feedback is not available. Therefore, teaching signals are calculated based on the predictions provided by the forward model. The error signal, at time t, for joint i is then given by $\epsilon_i(t) = (\theta_i(t) - \hat{\theta}_i(t))$, with $\theta_i(t)$ denoting the desired position of joint i at time t and $\hat{\theta}_i(t)$ its predicted position estimated by the corresponding cerebellar forward model. When forward predictions are not available (i.e. when the DCN activity of corresponding microcomplex are at baseline), then the input-output function stored in the inverse model must be preserved. Hence, heterosynaptic LTD (Eq. A10) and homosynaptic LTP (Eq. A9) at PF–PC synapses must lead to a homeostatic condition (see Sec. S1.7))

Teaching signal for the forward predictor model

For the forward model, the teaching signal relies on the perceived state of each limb. The mean firing rates of IO cells in each microcomplex vary according to a set of radial basis functions spanning the state space uniformly. For instance, in the microcomplex sub-serving the prediction of the shoulder angular position (Fig. S4), the mean firing rates of each IO cell i varies as:

$$r_i(t) = k \cdot \exp\Big(- \frac{(\theta - \theta_i)^2}{2\sigma_i^2} \Big) \tag{A14}$$

where $k = 10$ is a scaling factor, θ_i is the "preferred angle" of the cell, and σ determines the degree of overlap between adjacent IO responses. In this microcomplex, a group of 40 IO cells shares the same preferred angle. Each group of IO neurons targets two distinct PCs, which in turn inhibit the same DCN unit. The latter codes for the same portion of the θ state space (and has the same preferred angle θ_i) than the IO cells that modulate its inhibitory PC afferents. According to Eq. A14 and to the plasticity rules (see also example in Fig.. S4):

- If the firing rate of the group of IO cells with preferred angle θ_i is $r_i(t) \sim 1$ Hz, then LTD and LTP at PF–PC synapses of the two PCs driven by these two IO cells compensate each other. No learning occurs.

- If the firing rate of the IO cells with preferred angle θ_i is $1 < r_i(t) \leq 10$ Hz, then LTD dominates LTP at the PF–PC synapses of the two PCs driven by these two IO cells. Thus, over training, the DCN unit whose preferred angle is close to θ_i tends to increase its firing activity, whereas the other DCN units tend to either decrease or maintain their spike frequency. As a consequence, the decoding scheme used to readout the population activity of DCN neurons in the forward predictor model (Eq. A7) will tend towards an estimate of the next angular position close to θ_i.
- Conversely, if the firing rate of the IO cells with preferred angle θ_i is $0 \leq r_i(t) < 1$ Hz, then LTP dominates and the corresponding DCN neuron tends to decrease its spike frequency. Thus, this DCN unit will not contribute to the population decoding scheme significantly.

During *offline consolidation*, the real state of each limb is not perceived by the system (sensory feedback isn't available), hence no modification occurs in forward microcomplexes, and $r_i(t) = 1$ Hz for all IO neurons.

S1.7 Bistability of the forward model

We assume that forward predictions are useful only when accurate (Shadmehr et al., 2010). The accuracy of the forward prediction is reflected by a bistable behaviour, with active DCN neurons in the presence of reliable predictions and with below-threshold DCN activity otherwise. This all-or-none response results from the connectivity and coding schemes employed for the forward model. Recall that each DCN unit and each IO cell codes for a specific value of a variable, with the whole range of values being distributed over the entire population of cells.

The following example illustrates how the all-or-none response of the forward model works. Before training, DCN neurons discharge at baseline activity due to dominant PC inhibition —which results from the initialised large synaptic weights between PFs and PCs. When feeding this "naïve" network with a torque command τ_1', the latter activates a subset of GGs (G_1), which in turn excite the PCs, and then inhibit DCN activity. Let us now suppose that the actual execution of τ_1' took the shoulder at an angular position θ, and let IO_θ be the subset of IO cells coding for that angular value. The discharge of IO_θ cells triggers LTD between active PFs and PCs. Let PC_θ denote the subgroup of PCs targeted by IO_θ. The induction of LTD decreases the connection strength between G_1 and the PC_θ. Then, the activity of PC_θ directly influences the activity of the subgroup of DCN cells that code for the angular position θ, i.e. DCN_θ. Reiterating this adaptation process will incrementally decrease the activity of the PC_θ population and prevent it from inhibit DCN_θ cells completely. Hence, in response to the next presentation of the torque command τ_1', DCN_θ cells will discharge and the output of the forward model will code for the predicted angular position θ. Conversely, if we now present a new motor command τ_2', a new set G_2 of GCs will become active. Since previous training did not modify the connections between G_2 and PCs, DCN cells will remain silent (due to dominant PC inhibition).

This bistability is fundamental in our simulations for two reasons. First, during online processing, the simulated high level controller uses incoming signals from forward models to recompute the optimal trajectory. Thus, erroneous state predictions conveyed by the forward models would lead to catastrophic

performance. Second, during offline processing, the predictive outputs of forward models is exploited to extract the teaching signal for the inverse corrector models. Again, suboptimal state predictions would favour destructive interferences.

S1.8 Sparseness of neural activity

We quantify both population and lifetime sparseness (Willmore and Tolhurst, 2001; Martinet et al., 2011) of simulated MF and GC activity by means of the *kurtosis* function (Field, 1994).

Population kurtosis is defined as:

$$K(s) = \left\langle \left[\frac{r_j(s) - \bar{r}_J(s)}{\sigma_J(s)} \right]^4 \right\rangle_{j \in J} - 3 \tag{A15}$$

with $\bar{r}_J(s)$ and $\sigma_J(s)$ representing the mean and the standard deviation of the population activity distribution for a given input s, respectively. It estimates how many neurons j in a population J are, on average, simultaneously responding to an input s.

Single cell lifetime kurtosis is defined as:

$$K(j) = \left\langle \left[\frac{r_j(s) - \bar{r}_j}{\sigma_j} \right]^4 \right\rangle_{s \in S} - 3 \tag{A16}$$

with $\bar{r}_j$ and σ_j being the mean and the standard deviation of the cell response r_j to the set of inputs S, respectively. It assesses how rarely over time a neuron j responds to inputs S.

To compute the Kurtosis values reported in the main Results section, we fed the cerebellar networks with a set of 10000 distinct patterns covering the input entry space uniformly. Each pattern was considered independent from the others and presented 100 times for 100 ms.

S1.9 LTD–LTP compensation and stabilisation protocol

In order to evaluate the extent of LTD–LTP compensation at parallel fibre–Purkinje cell synapses, we used a simple stabilisation protocol in which the input/output relationship of the cerebellar microcomplex model had to remain stable over time. The protocol consisted of one session of 1000 s during which we fed a subset of 100 mossy fibres with a constant current making them discharge at approximately 100 Hz. A null error signal was used to modulate the firing rate $r(t)$ of each IO cell. Thus, each IO cell discharged at approximately 1 Hz following a Poisson process (we recall that 1 Hz is the firing rate of IO cell when no error is sensed). We measured the variability of simulated DCN activity by computing the coefficient of variation (CV_{DCN}) as a function of the number of PCs projecting onto each DCN unit. We averaged the results over a population of 10 model instances (see Fig. 4 F).

S1.10 Computational aspects of the model implementation

The spiking neurons of the cerebellar network were simulated by using a table-based event-driven simulator, namely the EDLUT simulator (Ros et al., 2006). The control system was tested in simulations

on a Intel Centrino dual core –2.4 GHz. Each cerebellar network consisted of nearly 40000 neurons and approximately 3500000 synaptic connections. During 1 s of simulation, the cerebellar network received an average of 2250 spikes, delivered 3350 output spikes, and processed approximately 2500000 events. Under these conditions the simulator ran at 1/2 real time speed.

Model of the controller

S2.1 Minimum jerk model

We use a minimum-jerk model (Flash and Hogan, 1985) to determine the optimal trajectory of the hand between an initial position and a target, as it is done in Carrillo et al., 2008. This method helps to to reduce the computational load and the degree of freedom of a given movement, and it constrains the limbs to act as one unit instead of sets of muscles and joints.

Jerk is defined as the rate of change (i.e. derivative) of acceleration along time. If $x(t)$ is a function defining the position of a moving object along time t, then jerk is defined by: $x^{(3)}(t)$. The minimum jerk model is an optimization methods based on smoothness cost function defined on the whole movement (from time t_0 to time t_1):

$$C(x) = \frac{1}{2} \int_{t_0}^{t_1} \left[x^{(3)}(t) \right]^2 dt \tag{A17}$$

We use calculus of variation methods to determine the function X_j that minimizes C.

Any function $y(t)$ describing a movement with the same boundary conditions (initial and final position, velocity and acceleration) can be written as:

$$y(t) = X_j(t) + e\eta(t) \tag{A18}$$

where e is a real number and η a function satisfying the following boundary conditions:

$$\begin{array}{ccccc} \eta(t_0) & = & \eta(t_1) & = & 0 \\ \dot{\eta}(t_0) & = & \dot{\eta}(t_1) & = & 0 \\ \ddot{\eta}(t_0) & = & \ddot{\eta}(t_1) & = & 0 \end{array} \tag{A19}$$

As X_j represents the minimum C, we should have:

$$\int_{t_0}^{t_1} X_j^{(3)}(t)\eta^3(t)dt = 0 \tag{A20}$$

for all functions η. Given the boundary values of η, integrating by parts the left side of the above equation leads to:

$$\int_{t_0}^{t_1} X_j^{(6)}(t)\eta(t)dt = 0 \tag{A21}$$

This must hold true for each function η, therefore $X_j^{(6)}(t) = 0$, $\forall t \in [t_0, t_1]$:

If we now consider a two-dimensional movement $X(t) = (x(t), y(t))^t$, the previous demonstration still holds and the result can be projected onto each dimension:

$$\forall\, t \in [t_0, t_1],\; x_j^{(6)}(t) = 0 \; and \; y_j^{(6)}(t) = 0 \tag{A22}$$

S2.2 Inverse kinematics model

The inverse kinematics of the arm are given by the following set of equations.

$$\begin{aligned} \theta_s &= atan\left(\frac{y}{x}\right) - atan\left(\frac{l_2 \sin\theta_e}{l_1 + l_2\cos\theta_e}\right) \\ \theta_e &= acos\left(\frac{x^2+y^2-l_1^2-l_2^2}{2l_1 l_2}\right) \end{aligned} \tag{A23}$$

$$\begin{aligned} \begin{pmatrix} \dot\theta_s \\ \dot\theta_e \end{pmatrix} &= J^{-1} \begin{pmatrix} \dot x \\ \dot y \end{pmatrix} \\ \begin{pmatrix} \ddot\theta_s \\ \ddot\theta_e \end{pmatrix} &= J^{-1} \left[\begin{pmatrix} \ddot x \\ \ddot y \end{pmatrix} + \begin{pmatrix} l_1\dot\theta_s^2 \cos\theta_s + l_2(\dot\theta_s + \dot\theta_e)^2 \cos(\theta_s+\theta_e) \\ l_1\dot\theta_s^2 \sin\theta_s + l_2(\dot\theta_s + \dot\theta_e)^2 \sin(\theta_s+\theta_e) \end{pmatrix} \right] \\ J &= \begin{pmatrix} -l_1\sin\theta_s - l_2\sin(\theta_s+\theta_e) & -l_2\sin(\theta_s+\theta_e) \\ l_1\cos\theta_s + l_2\cos(\theta_s+\theta_e) & l_2\cos(\theta_s+\theta_e) \end{pmatrix} \end{aligned} \tag{A24}$$

J represents the Jacobian of the coordinates transformation, l_1 is the length of the upper arm and l_2 is the length of the forearm. We assume homogeneity along the limb, then $I_1 = (\frac{m_1}{3} + M_2)l_1^2$ and $I_2 = \frac{m_2 l_2^2}{3}$, where m corresponds to the weight of the limb, M for the weight of its control motor, and I its inertia at joint (again, subscript 1 represent the variables related to the upper arm and subscript 2 the variable related to the forearm). Parameters used for the arm are: $l_1 = 22.5\ cm$, $l_2 = 25.5\ cm$, $m_1 = 0.15\ kg$, $m_2 = 0.15\ kg$, $M_2 = 0.3\ kg$ as in Carrillo et al. (2008).

θ_s represents the angle of the upper arm relative to the X axis (shoulder angle), and θ_e the angle of the forearm relative to the upper arm (elbow angle). x and y correspond to the Cartesian coordinates of the arm end-point E.

S2.3 Inverse dynamics model

The inverse dynamics model of the arm is given by:

$$\begin{pmatrix} \tau_s \\ \tau_e \end{pmatrix} = M(\Theta_{des}) \begin{pmatrix} \ddot\theta_{s,des} \\ \ddot\theta_{e,des} \end{pmatrix} + C\left(\Theta_{des}, \dot\Theta_{des}\right) \begin{pmatrix} \dot\theta_{s,des} \\ \dot\theta_{e,des} \end{pmatrix} \tag{A25}$$

where τ_i is the torque to apply at joint i to follow the desired trajectory:
$T_{des}^{arm} = \left\{ \left(\Theta_{des}(t), \dot{\Theta}_{des}(t), \ddot{\Theta}_{des}(t) \right), t \in [0, \Delta T] \right\}$,

The matrices of inertia and of Coriolis and centrifugal coefficient are given by:

$$\begin{aligned} M(\Theta) &= \begin{pmatrix} I_1 + I_2 + 2m_2 l_1 l_{g2} \cos\theta_e + m_2 l_1^2 & I_2 + m_2 l_1 l_{g2} \cos\theta_e \\ I_2 + m_2 l_1 l_{g2} \cos\theta_e & I_2 \end{pmatrix} \\ C(\Theta, \dot{\Theta}) &= \begin{pmatrix} -2m_2 l_1 l_{g2} \dot{\theta}_e \sin\theta_e & -m_2 l_1 l_{g2} \dot{\theta}_e \sin\theta_e \\ m_2 l_1 l_{g2} \dot{\theta}_s \sin\theta_e & 0 \end{pmatrix} \end{aligned} \tag{A26}$$

where l_{g2} is the distance of the center of mass of the forearm relative to the elbow. In our simulation, we suppose a uniform repartition of the mass of the limb, then $l_{g2} = \frac{l_2}{2}$.

References

Arenz, A., R. A. Silver, A. T. Schaefer, and T. W. Margrie (2008). The contribution of single synapses to sensory representation in vivo. *Science (80-) 321*(5891), 977–80.

Boyden, E. S., A. Katoh, and J. L. Raymond (2004). Cerebellum-dependent learning: the role of multiple plasticity mechanisms. *Annu Rev Neurosci 27*(1), 581–609.

Carrillo, R. R., E. Ros, C. Boucheny, and O. J. D. Coenen (2008). A real-time spiking cerebellum model for learning robot control. *Biosystems 94*(1-2), 18–27.

Chadderton, P., T. W. Margrie, and M. Häusser (2004). Integration of quanta in cerebellar granule cells during sensory processing. *Nature 428*(6985), 856–860.

D'Angelo, E. and C. I. De Zeeuw (2009). Timing and plasticity in the cerebellum: focus on the granular layer. *Trends Neurosci 32*(1), 30–40.

De Zeeuw, C. I. and C. H. Yeo (2005). Time and tide in cerebellar memory formation. *Curr Opin Neurobiol 15*(6), 667–74.

Dean, P., J. Porrill, C.-F. Ekerot, and H. Jörntell (2010). The cerebellar microcircuit as an adaptive filter: experimental and computational evidence. *Nat Rev Neurosci 11*(1), 30–43.

Desmond, J. E. and J. W. Moore (1988). Adaptive timing in neural networks: the conditioned response. *Biol Cybern 58*(6), 405–415.

Eccles, J. C., M. Ito, and J. Szentágothai (1967). *The Cerebellum as a Neuronal Machine*, Volume 6. Springer-Verlag.

Field, D. J. (1994). What is the goal of sensory coding? *Neural Comput 6*(4), 559–601.

Flash, T. and N. Hogan (1985). The coordination of arm movements: an experimentally confirmed mathematical model. *J Neurosci 5*(7), 1688–1703.

Gibson, A., K. Horn, and M. Pong (2004). Activation of climbing fibers. *The Cerebellum 3*(4), 212–221.

Hansel, C., D. J. Linden, and E. D'Angelo (2001). Beyond parallel fiber LTD: the diversity of synaptic and non-synaptic plasticity in the cerebellum. *Nat Neurosci 4*(5), 467–475.

Hirano, T., D. Watanabe, S. Kawaguchi, I. Pastan, and S. Nakanishi (2002). Roles of inhibitory interneurons in the cerebellar cortex. *Ann N Y Acad Sci 978*, 405–412.

Ito, M. (2006). Cerebellar circuitry as a neuronal machine. *Prog Brain Res 78*, 272–303.

Ito, M. and M. Kano (1982). Long-lasting depression of parallel fiber-Purkinje cell transmission induced by conjunctive stimulation of parallel fibers and climbing fibers in the cerebellar cortex. *Neurosci Lett 33*(3), 253–258.

Jörntell, H. and C.-F. Ekerot (2006). Properties of somatosensory synaptic integration in cerebellar granule cells in vivo. *The Journal of Neuroscience: The Official Journal of the Society for Neuroscience 26*(45), 11786–97.

Lamont, M. (2009). An electrophysiological analysis of deep cerebellar nuclei, with particular focus on Kv3 channels. *Bioscience Horizons 2*(1), 55–63.

Lev-Ram, V., S. T. Wong, D. R. Storm, and R. Y. Tsien (2002). A new form of cerebellar long-term potentiation is postsynaptic and depends on nitric oxide but not cAMP. *Proc Natl Acad Sci U S A 99*(12), 8389–8393.

Martinet, L.-E., D. Sheynikhovich, K. Benchenane, and A. Arleo (2011). Spatial learning and action planning in a prefrontal cortical network model. *PLoS Comput Biol 7(5): e1002045.*

Palkovits, M., E. Mezey, J. Hámori, and J. Szentágothai (1977). Quantitative histological analysis of the cerebellar nuclei in the cat. I. Numerical data on cells and on synapses. *Exp Brain Res 28*(1-2), 189–209.

Pugh, J. R. and I. M. Raman (2009). Nothing can be coincidence: synaptic inhibition and plasticity in the cerebellar nuclei. *Trends Neurosci 32*(3), 170–7.

Raman, I. M. and B. P. Bean (1999). Properties of sodium currents and action potential firing in isolated cerebellar purkinje neurons. *Ann N Y Acad Sci 868*, 93–96.

Rancz, E., T. Ishikawa, I. C. Duguid, P. Chadderton, S. Mahon, and M. Häusser (2007). High-fidelity transmission of sensory information by single cerebellar mossy fibre boutons. *Nature 450*(7173), 1245–8.

Ros, E., R. Carrillo, E. M. Ortigosa, B. Barbour, and R. Agas (2006). Event-driven simulation scheme for spiking neural networks using lookup tables to characterize neuronal dynamics. *Neural Comput 18*(12), 2959–2993.

Safo, P. and W. G. Regehr (2008). Timing dependence of the induction of cerebellar LTD. *Neuropharmacology 54*(1), 213–218.

Sejnowski, T. J. (1977). Storing covariance with nonlinearly interacting neurons. *J Math Biol 4*(4), 303–321.

Shadmehr, R., M. a Smith, and J. W. Krakauer (2010). Error correction, sensory prediction, and adaptation in motor control. *Annu Rev Neurosci 33*, 89–108.

Willmore, B. and D. J. Tolhurst (2001). Characterizing the sparseness of neural codes. *Network 12*, 255–270.

Yamazaki, T. and S. Tanaka (2007). A spiking network model for passage-of-time representation in the cerebellum. *Eur J Neurosci 26*(8), 2279–92.

Supplementary Results

Properties of cerebellar internal models subserving sensorimotor adaptation

S1.1 Sparse coding of contextual information by the cerebellar granular layer

Cerebellar granule cells (GCs) are thought to optimally re-encode mossy fibre (MF) inputs to enhance learning capacity at parallel fibre–Purkinje cell (PF–PC) synapses (Marr, 1969; Schweighofer et al., 2001; Brunel et al., 2004; Philipona and Coenen, 2004; Ito, 2006). Accordingly, our simulated granular layer produced a sparse representation (both in space and time) of the afferent MF signals (Figs. A1 A, B). A comparative kurtosis analysis (Field, 1994; Willmore and Tolhurst, 2001) of simulated GC and MF codes showed a significant increase of sparseness at the level of both population and unitary GC responses (Fig. A1 C). The population sparseness of the GC code was significantly larger relative to MF inputs ($K_{GC} = 14.1 \pm 1.8$, mean $\pm$ std; $K_{MF} = 4 \pm 2$; ANOVA, $F_{1,18} = 140.83$, $P < 0.001$), suggesting a better separation of sensorimotor contexts downstream from the GC layer. The selectivity of single neurons with respect to afferent patterns (i.e. lifetime kurtosis) was also significantly larger in GCs compared to MFs ($K'_{GC} = 25.7 \pm 2$; $K'_{MF} = 3.9 \pm 1.6$; ANOVA, $F_{1,18} = 710.08$, $P < 0.001$), suggesting that, on average, single GCs responded to a smaller number of contexts than single MFs.

S1.2 Bistability of forward predictor models

In our cerebellar model, the accuracy of a prediction was reflected by a bistable behaviour, with active DCN neurons (output) in the presence of reliable predictions, and with below-threshold DCN activity otherwise. This all-or-none response resulted from the connectivity and coding schemes employed for the forward model (see Supplementary Methods). When forward models were inactive, the mean prediction errors were large (e.g. larger than 20° for the predicted positions of both arm joints, Fig. A1 D). By contrast, when the forward models became active, the mean prediction errors reduced below 4° for both joints, with the prediction accuracy tending to improve with the increase of DCN spike rates. The number of context presentations necessary for FMs to reliably predict the shoulder and elbow joint angular positions was 5 ± 0.9 (mean $\pm$ s.e.) and 5.5 ± 0.7, respectively (Fig. A1 E). Similarly, few presentations were necessary to acquire accurate joint speed predictions for the two joints (8.1 ± 0.5 and 7.5 ± 0.5, respectively, Fig. A1 E).

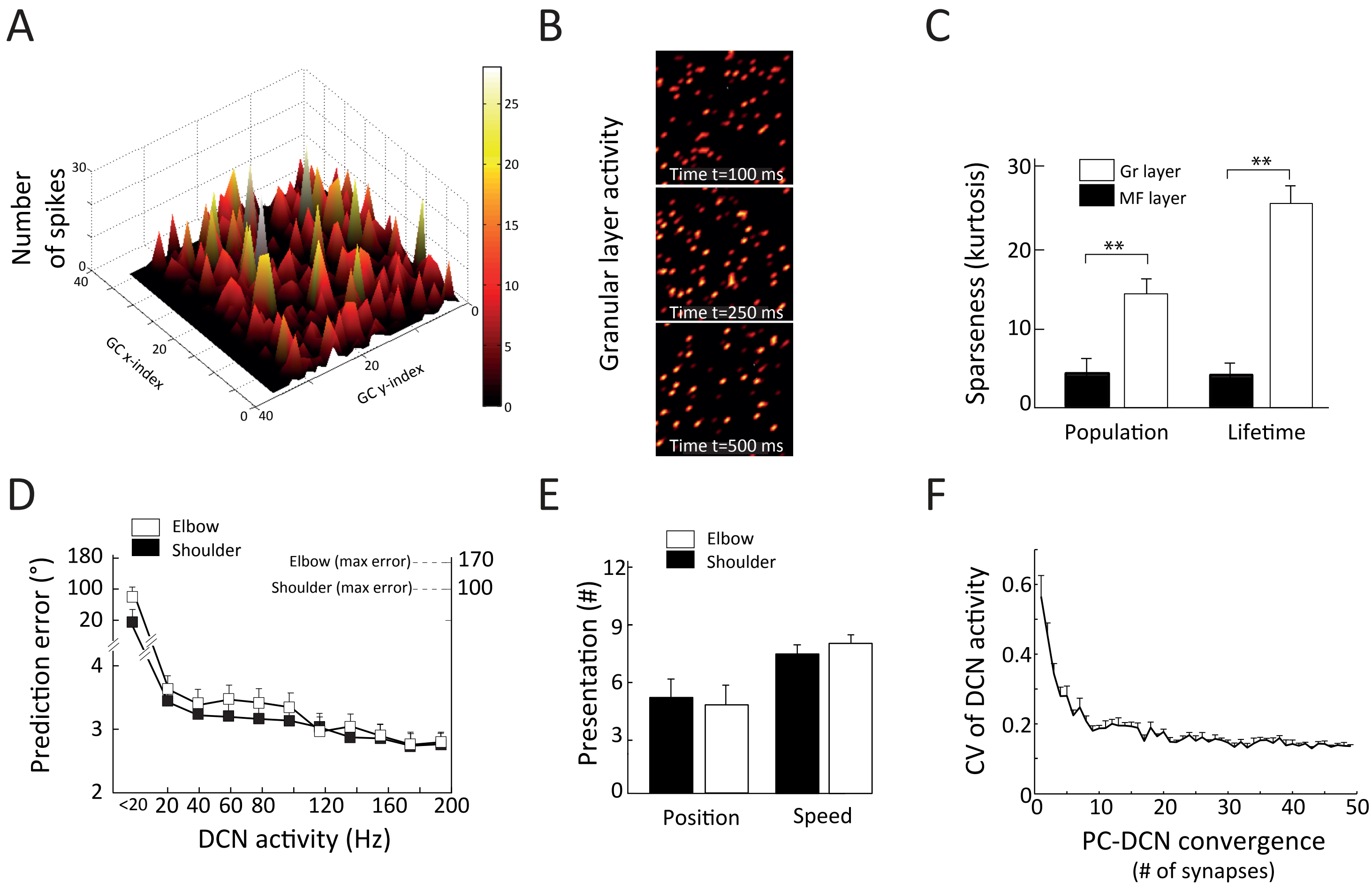

Figure A1. Properties of cerebellar internal models subserving sensorimotor adaptation. A. Global activity of the granular cells (GCs) of a forward model during a 700 ms simulation. The GC population is represented on a square array of 35 by 35 cells. The z axis shows the number of spikes that the neuron of index (i, j) fired during the generation of the movement. Every $10ms$, an average of 35 ± 1 GR cells discharged, which represents nearly 3% of the granular layer population and 7% of the active cells during the whole movement. **B.** Snapshots of GC population activity at three different times of the same trajectory (i.e. at time t=100; 200 and 500 ms). The same colour map of A is used. **C.** Comparison between population and lifetime kurtosis of GC and mossy fibre (MF) activity. **D.** Mean angular prediction error of the forward model activity for elbow and shoulder joints as a function of the DCN activity of the corresponding forward model. **E.** Mean number of presentations necessary to create stable context-response associations. **F.** Coefficient of variation of DCN activity as a function of the number of Purkinje cells (PCs) connected to each DCN.

S1.3 The amount of synaptic convergence from PCs onto DCNs is relevant to the stability of sensorimotor learning

The teaching signal driving learning in cerebellar internal models could happen to be unavailable or nil (e.g. during offline consolidation, see below). In this situation, previously learnt sensorimotor associations had to persist unchanged to avoid destructive interference. This requirement implied that, in the absence of a teaching signal, LTP and LTD at model PF–PC synapses had to compensate for each other, letting the cerebellar input-output dynamics unchanged. Maintaining this balance was not trivial to achieve because LTP and LTD are not symmetrical processes. Indeed, LTP at PF–PC synapses is an homosynaptic process occurring before knowing whether the teaching signal will be available or not. By contrast, LTD is a heterosynaptic mechanism triggered by climbing fibre activity and signaling past events (see Supplementary Methods).

In our simulations, we observed that LTP/LTD mutual compensation at PF–PC synapses depended on the connectivity layout of the cerebellar network, and in particular on the PC–to–DCN synaptic convergence. We assessed the extent of LTP/LTD compensation indirectly, by measuring the firing variability of simulated DCN cells. We quantified the coefficient of variation of DCN neurons (CV_{DCN}) as a function of the number of PCs projecting onto each DCN unit (Fig. A1 F; see Supplementary Methods for protocol details). The CV_{DCN} was large for small PC-to-DCN synaptic convergence (e.g. < 5 PC afferent projections per DCN cell), whereas it significantly decreased for larger values of the PC-to-DCN convergence (e.g. ≥ 30 PC synaptic contacts per DCN neuron). This result is consistent with neuroanatomical data suggesting that each DCN cell is likely to integrate information from tenth to hundreds PCs (Palay, 1974; Palkovits et al., 1977).

References

Brunel, N., V. Hakim, P. Isope, J.-P. Nadal, and B. Barbour (2004). Optimal information storage and the distribution of synaptic weights: perceptron versus Purkinje cell. *Neuron 43*(5), 745–757.

Field, D. J. (1994). What is the goal of sensory coding? *Neural Comput 6*(4), 559–601.

Ito, M. (2006). Cerebellar circuitry as a neuronal machine. *Prog Brain Res 78*, 272–303.

Marr, D. (1969). A theory of cerebellar cortex. *J Physiol 202*(2), 437–470.

Palay, S. L. (1974). *Cerebellar cortex: cytology and organization.* (Berlin, Heidelberg, New York): Springer.

Palkovits, M., E. Mezey, J. Hámori, and J. Szentágothai (1977). Quantitative histological analysis of the cerebellar nuclei in the cat. I. Numerical data on cells and on synapses. *Exp Brain Res 28*(1-2), 189–209.

Philipona, D. and O. J.-M. D. Coenen (2004). Model of granular layer encoding in the cerebellum. *Neurocomputing 58*, 575–580.

Schweighofer, N., K. Doya, and F. Lay (2001). Unsupervised learning of granule cell sparse codes enhances cerebellar adaptive control. *Neuroscience 103*(1), 35–50.

Willmore, B. and D. J. Tolhurst (2001). Characterizing the sparseness of neural codes. *Network 12*, 255–270.

Supplementary Figures

Figure Captions

Figure S1 Coupling scheme for online and offline sensorimotor adaptation. **A. Online learning scheme**. At each time step, the limb controller (motor cortex) receives the desired state in the form of target position of the arm end-point and transforms them into torque motor commands for each joint. The desired state is also sent to a set of cerebellar inverse corrector models (IMs) that calculate context-dependent adjustments of motor commands prior to their transmission to the motor apparatus (e.g. muscles). An efference copy of final motor commands is sent to a set of cerebellar forward predictor models (FMs) that estimate the future state of each joint. Based on FM predictions, the controller can then eventually update the inverse dynamics calculations. At each time step, the teaching signal for the IM is computed by sensing the difference between desired and actual state, whereas the teaching signal for the FM is based on the sensed actual state only. **B. Offline learning scheme**. In the absence of sensory feedback during offline processing, the state predictions of at least partially trained FMs can be used to train the IMs offline —by assuming that the entire sequence of actions executed during online training can be replayed offline, in order to provide an input to the controller.

Figure S2 Coding scheme for the inverse corrector implemented by the cerebellar microcomplex model. Example of error encoding and output decoding for the virtual agonist muscle of the shoulder joint (positive correction). The teaching signal encodes the angular error, that is the difference between desired θ_s and actual $\bar{\theta}_s$ angular position of the shoulder. Here, the error $\theta_s - \bar{\theta}_s$ indicates that the torque command sent to the shoulder must increase, i.e. $[\epsilon_i^+] > 0$. Then the mean firing rate of IO neurons is set to $r_{\tau_s^+}(t) = 10$ Hz, which makes LTD to take over LTP in the active PF–PC synapses of the corresponding microcomplex. The consequent decrease of PF–PC efficacy reduces the inhibitory action of PCs onto DCN neurons. Hence, the next time that the microcomplex will receive the same contextual input, the average population activity of DCN neurons $\langle\nu(t)\rangle$ will increase, reinforcing the correction signal $\tau_s^+(t)$.

Figure S3 Coding scheme for the forward predictor implemented by the cerebellar microcomplex model. Example of error encoding and output decoding for the predicted angular position of the shoulder. The teaching signal encodes the actual position $\bar{\theta}_s$ reached by the shoulder joint 100 ms after the execution of the last motor command. The firing rates of IO cells $r_i(t)$ vary according to a set of radial basis functions spanning the $\bar{\theta}_s$ state space uniformly. A group of IO cells share the same preferred angle and each group targets two distinct PCs, which in turn inhibit a single DCN unit. The latter codes for the same portion of the angular state space (and has the same preferred angle θ_i) than

the two IO cells that modulate its inhibitory PC afferents. Depending on the firing rate of a group of two IO cells, three cases can be distinguished: *(i)* if the firing rate of the IO cells with preferred angle θ_i is $r_i(t) \sim 1$ Hz (r_1 in this example), then LTD and LTP at PF–PC synapses of the two PCs driven by these two IO cells compensate each other and no learning occurs. The corresponding DCN neuron tends to stabilise its spike frequency (ν_1); *(ii)* if the firing rate of the two IO cells with preferred angle θ_i is $0 \leq r_i(t) < 1$ Hz (r_{n-1} and r_n in this example), then LTP dominates and the corresponding DCN neuron tends to decrease its spike frequency (ν_{n-1} and ν_n); *(iii)* if the firing rate of the two IO cells with preferred angle θ_i is $1 < r_i(t) \leq 10$ Hz (r_2 in this example), then LTD dominates LTP at the PF–PC synapses of the two PCs driven by these two IO cells. Thus, over training, the DCN unit whose preferred angle is close to θ_i tends to increase its firing activity (ν_2 in this example). As a consequence, the decoding scheme used to readout the population activity of DCN neurons will tend towards an estimate of the next angular position $\hat{\theta}$ close to $\bar{\theta}_s$. (The colour code used to describe the intensity of neuronal discharges is the following: white for no activity, light blue for low activity, and dark blue for high activity).

Figure S4 **Adaptation in forward and inverse cerebellar models**. **A**. Normalised prediction error for the angular position of the shoulder in three sample contexts. The prediction error of forward cerebellar models (FMs) is large at the beginning of the training, but it decreases to small values after few motor command presentations. Simulated FMs have a bistable behaviour: at the beginning of training, their output is silent (i.e. DCN neural activity is at baseline) due to dominant inhibition from PCs. After a few input presentations, learning inhibits PCs enabling DCN firing, and the prediction error of FMs decreases abruptly. **B**. Time course of FM learning. After 60 presentations of the same context, simulated FMs could reliably predict the outcome of $\sim 35\%$ of torque commands applied to both joints —i.e. they could estimate both future angular position and velocity prior to the execution of 35% of the received torque commands. The performance of FM predictors increased monotonically and reached $65 - 70\%$ after 5 minutes of training (i.e. 300 presentations of the same target). **C**. Time course of inverse model (IM) adaptation. Learning in simulated IM correctors improved the accuracy of motor command execution. The residual error of IM correctors decreased steadily over time. The convergence of the learning process required ~ 300 presentations.

Figure S5 **Coupling forward and inverse models enhances online sensorimotor adaptation. Time course of the distance error**. The mean normalised distance error (averaged over all subjects) is plotted as a function of training trials. Only subjects using a coupled cerebellar model (i.e. CM group, plain green curves) succeeded in reducing the distance error effectively over training. Subjects using the

inverse model only (i.e. IM, red line) demonstrated a slower learning process compare to those using a forward model only (i.e. FM, blue line).

Figure S6 **Examples of torque commands over time**. The time course of torque intensities for target 1 and target 2 (green circles) are shown (top and bottom rows, respectively) through the realisation of one reaching movement ($\sim$ 700ms). For both targets, 4 different configurations are shown (from left to right: with no adaptation, forward model scheme only, inverse model scheme only, and coupling scheme). Note that the torques sent to each articulation are not static during movement. Rather, they change dynamically to achieve the desired trajectory. Also, two symmetrical movements imply two sets of independent torque commands (compare first and second trajectories, in top and bottom rows, when no adaptation is allowed). Finally, a correction of the angular deviation implies a new set of torque commands independent from those of the not corrected movement (compare torque commands of 'No adaptation scheme' versus 'Coupling scheme' for target 2).

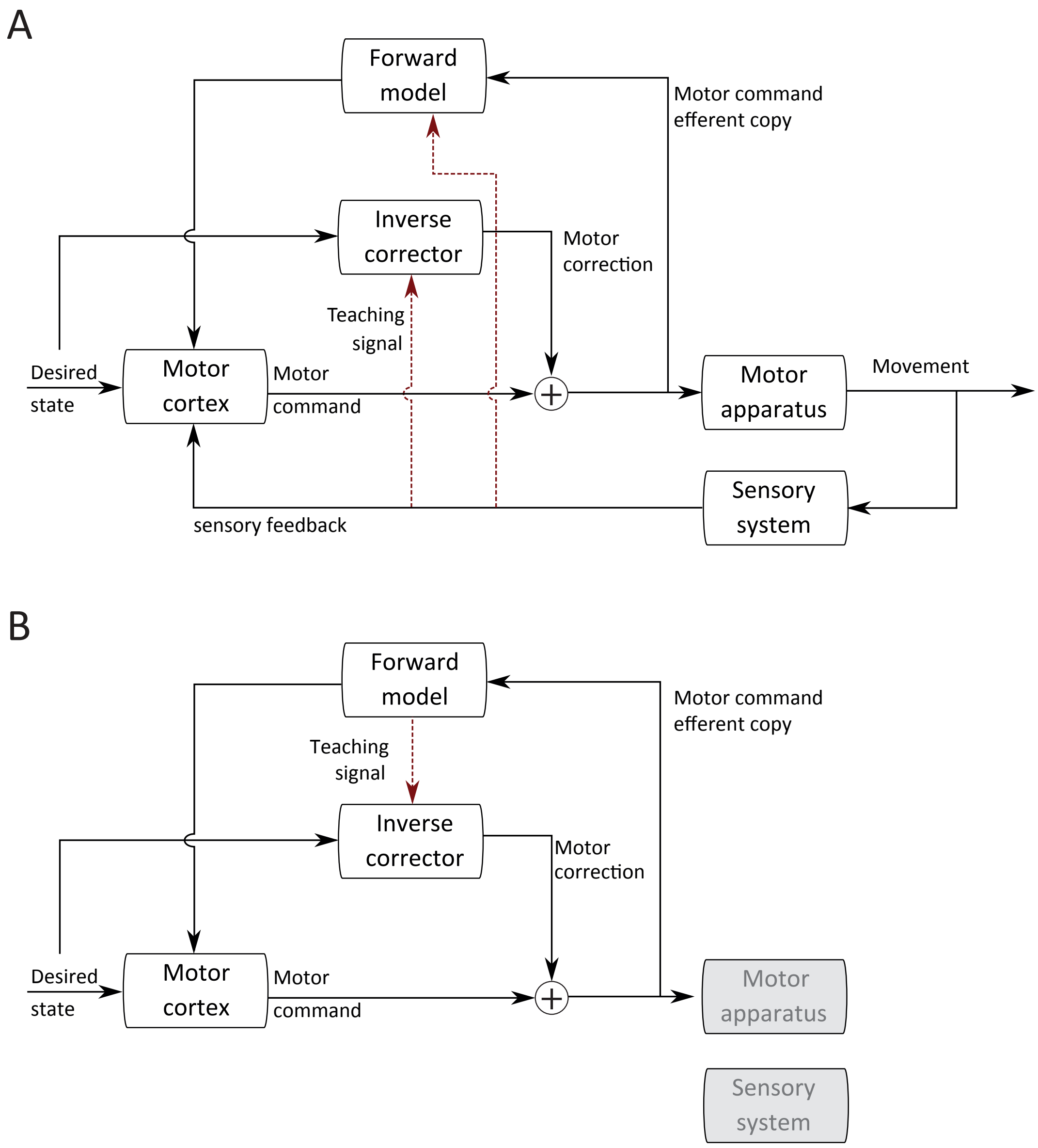
A
Forward model
Motor command efferent copy
Inverse corrector
Motor correction
Teaching signal
Desired state
Motor cortex
Motor command
Motor apparatus
Movement
Sensory system
sensory feedback
B
Forward model
Motor command efferent copy
Teaching signal
Inverse corrector
Motor correction
Desired state
Motor cortex
Motor command
Motor apparatus
Sensory system

Figure S1

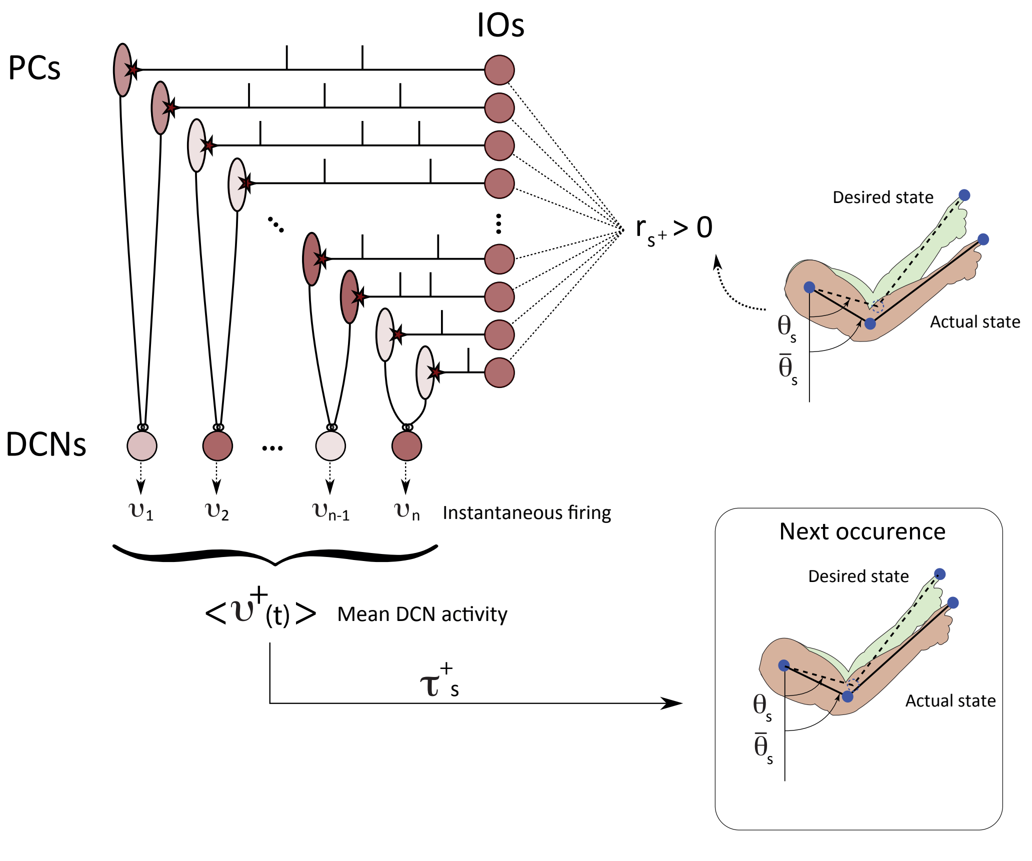
IOs
PCs
DCNs
$r_{s^+} > 0$
Desired state
Actual state
θ_s
$\bar{\theta}_s$
υ_1 υ_2 υ_{n-1} υ_n Instantaneous firing
$<\upsilon^+(t)>$ Mean DCN activity
τ^+_s
Next occurence
Desired state
Actual state
θ_s
$\bar{\theta}_s$

Figure S2

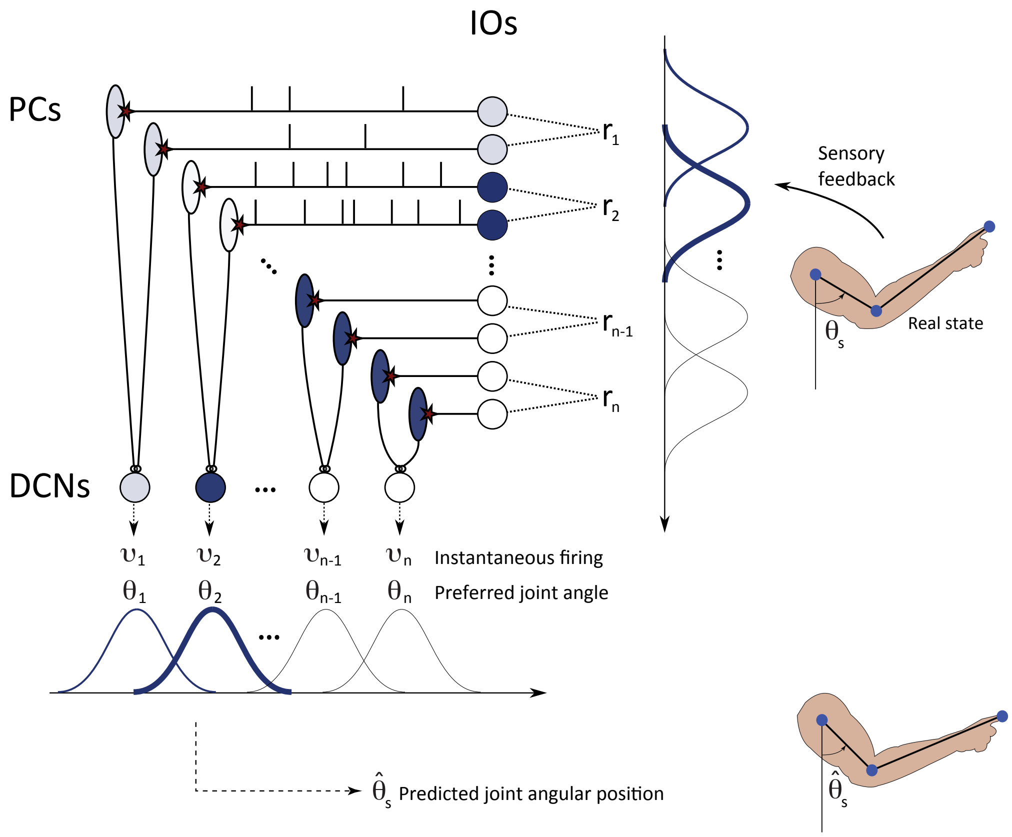
IOs
PCs
r1
r2
rn-1
rn
Sensory feedback
θs
Real state
DCNs
υ1 υ2 υn-1 υn Instantaneous firing
θ1 θ2 θn-1 θn Preferred joint angle
θ̂s Predicted joint angular position
θ̂s

Figure S3

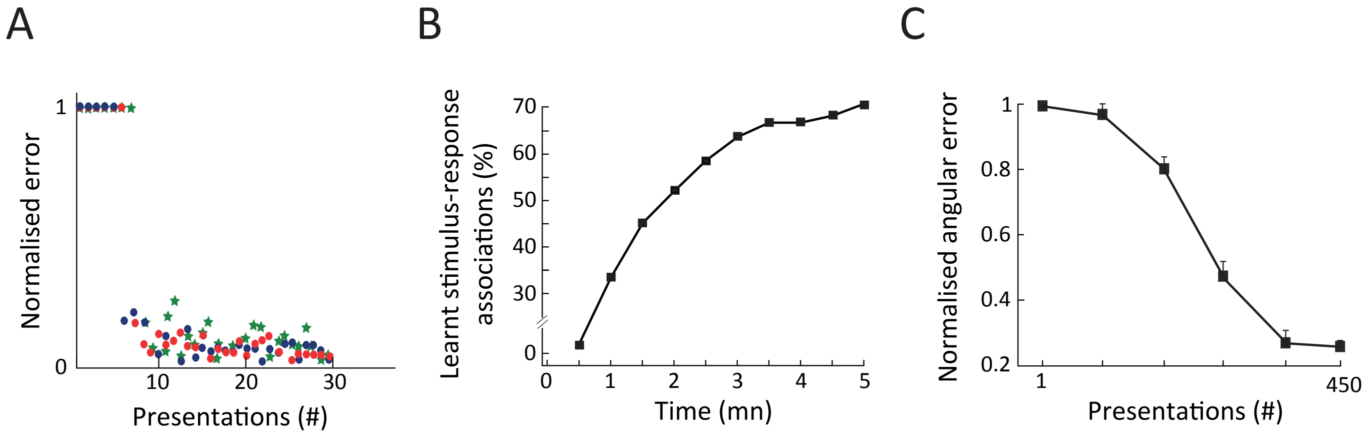
A
B
C
1
0
Normalised error
10
20
30
Presentations (#)
Learnt stimulus-response associations (%)
70
60
50
40
30
0
1
2
3
4
5
Time (mn)
Normalised angular error
1
0.8
0.6
0.4
0.2
1
450
Presentations (#)

Figure S4

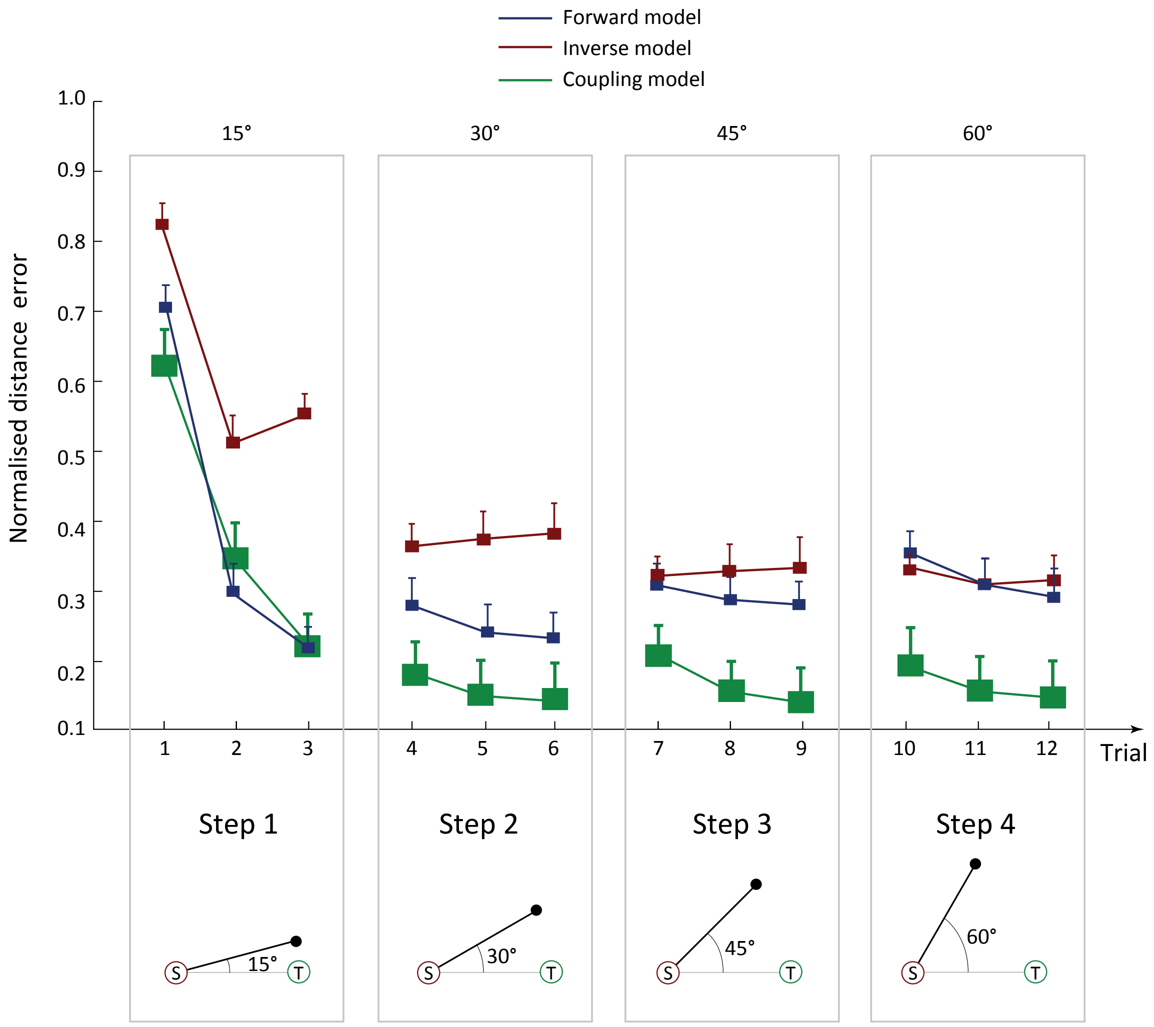
Forward model
Inverse model
Coupling model
Normalised distance error
1.0
0.9
0.8
0.7
0.6
0.5
0.4
0.3
0.2
0.1
15°
30°
45°
60°
1
2
3
4
5
6
7
8
9
10
11
12
Trial
Step 1
Step 2
Step 3
Step 4
S
T
15°
30°
45°
60°

Figure S5

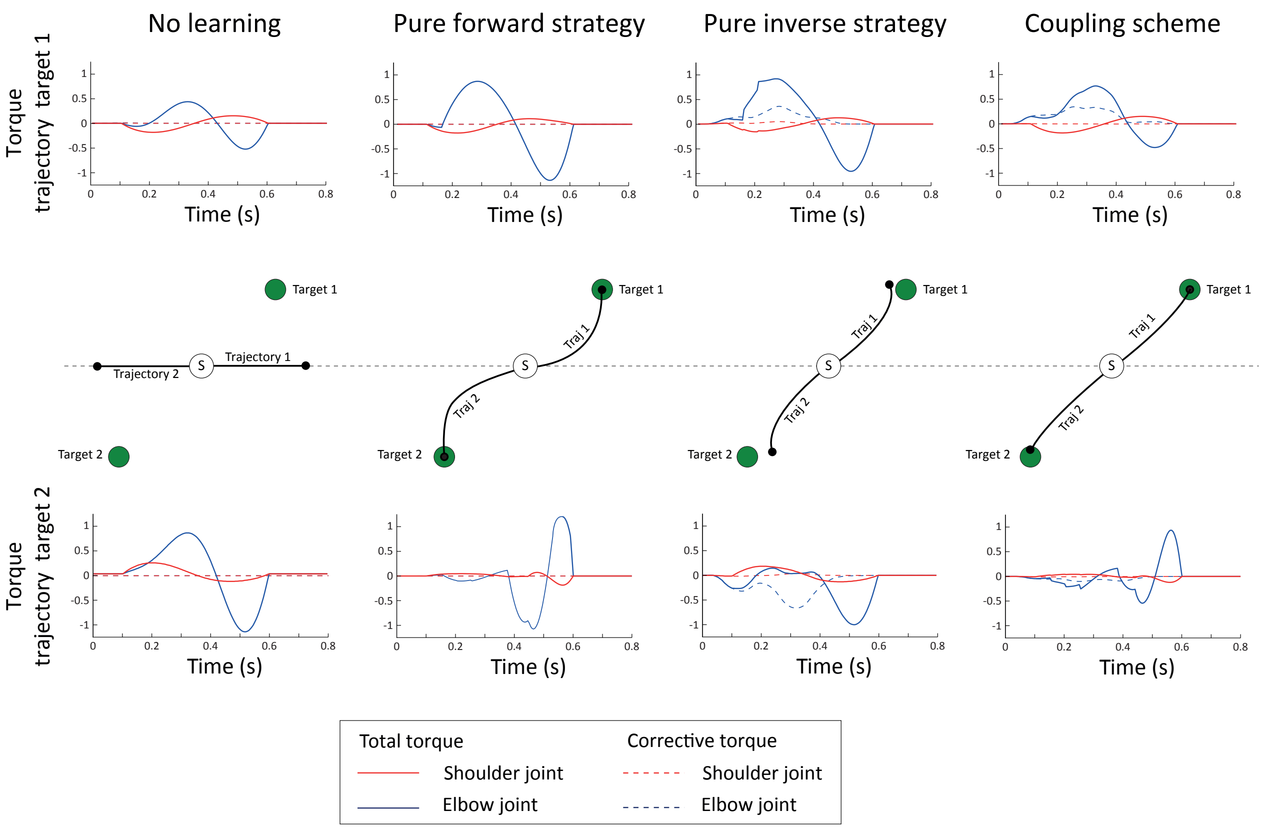
No learning
Pure forward strategy
Pure inverse strategy
Coupling scheme
Torque trajectory target 1
Time (s)
Target 1
Target 2
Trajectory 1
Trajectory 2
S
Traj 1
Traj 2
Torque trajectory target 2
Total torque
Corrective torque
Shoulder joint
Elbow joint

Figure S6
